# MAG-SOLex Molecular Representation: A Methodology for Handling Complex Molecules in Algorithms

**DOI:** 10.1021/acsomega.4c08940

**Published:** 2025-02-04

**Authors:** Diego Telles Fernandes, Karina Klock da Costa, Helton Siqueira Maciel, Radha Liliane Pinto Gonçalves, Dirceu Noriler

**Affiliations:** † PETROBRAS, Research and Development Center − CENPES, Av. Horacio de Macedo, 950, Ilha do Fundão, Rio de Janeiro, RJ 20031-912, Brazil; ‡ UNICAMP, School of Chemical Engineering, Albert Einstein Av, 500, Campinas 13083-872, Brazil

## Abstract

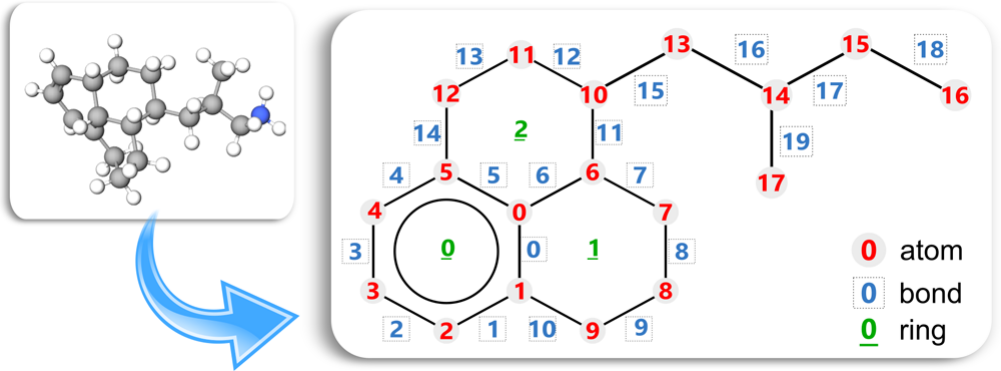

Different molecular representations exist to provide visual and mathematical means to depict complex structures. However, the detailing required for algorithmic applications remains a challenge. This study presents a methodology that uses two matrices to obtain a detailed molecular description. The proposed matrices are Molecule as a Graph (MAG), which describes a molecular graph providing detailed information about the bonds between atoms in a molecule, and an extended version of Structure-Oriented-Lumping (SOLex), representing each core in a molecule with 44 increments organized into vectors, capable of describing multicore structures. The combined use of these representations integrates the agility of SOLex with the structural detailing of MAG, enabling a rapid acquisition of detailed information about molecules. This can be applied in the calculation of thermodynamic properties using group contribution methods, as well as in the depiction of chemical reactions at molecular level, enabling precise identification of groups and reactive sites in molecules efficiently, even in complex algorithms. The methodology was designed for hydrocarbons from petroleum and biomass-derived sources, but can also be applied to different types of molecules, providing a versatile and efficient means of handling molecular information.

## 1 Introduction

Molecules are fundamental components that form the basis for understanding the composition of mixtures, reaction kinetics, thermodynamics, and property calculation in chemical processes. In representing a chemical compound, it is necessary to have a nomenclature that uses a reproducible notation capable of representing simple atoms to complex structures. Moreover, individual molecules or solutions of multiple ones require appropriate and mathematically manipulable representations for their application in algorithms and computational software.

Over time, as computational development progressed, representations have become increasingly concerned with the mathematical treatment of the molecules, reflecting a focus on facilitating mathematical manipulation within the broader concern for proper representation (Wiswesser^1^). However, as the complexity of molecules increases, the representation must be more detailed to predict, for example, isomers and types of bonds for a better estimate of the properties of the compound, especially when it is present in mixtures. Bjerrum^2^ presented a linear notation that represents the structure of chemical molecules using a sequence of alphanumeric characters and symbols called Simplified Molecular Input Line Entry System (SMILES). This allows for the description of a molecule’s composition and structure, including atoms, bonds, branches, and cyclic structures, in a compact and machine-readable format and its widely used.^3−5^

When analyzing mixtures, accurate representation of the molecules is essential for developing robust models that can provide a comprehensive understanding of complex chemical systems.^6^ Molecular models can be adapted according to the required application and they can include information on the molecular structure that allows not only for its description but also for manipulating this information to calculate mixture properties, describe chemical reactions, or achieve specific objectives.

In order to represent mixtures of molecules in algorithms, Quann and Jaffe^7^ at Mobil Research and Development Co. developed an approach called Structure-Oriented Lumping (SOL), which models the chemistry of complex mixtures based on the idea that hydrocarbon molecules can be described as a vector whose elements represent structural features that are sufficient to construct any molecule. This methodology and its variations have been applied in several works, such as Al Jamri et al.,^8^ Chen et al.,^9^ Chen et al.,^10^ Maciel,^11^ Chen et al.,^12^ and have shown good adaptation for use in algorithms, being adjustable according to the molecules of interest. For example, Nguyen et al.^13^ presented a comprehensive framework for creating a structure-based lumping kinetic model for refinery reactors, utilizing petroleomics data to accurately predict product composition and reaction behavior, with a specific application to hydrodesulfurization of light gas oil (LGO-HDS).

Due to its simplicity and ease of obtaining molecular information from the inspection of these vectors, this approach has been utilized within more complex and computationally expensive algorithms, such as molecular reconstructions and the development of reaction models.^6,7,11,14^ However, despite its accessible computational handling, the SOL methodology may lose molecule-specific information, as it does not allow for detailed description of the molecules. For example, it is not possible to differentiate isomers or precisely know how atoms are bonded, which hinders the development of reaction models and the application of more detailed group contribution methods, such as that of Marrero and Gani.^15^

Another approach to representing a chemical compound is through a bond-electron matrix (BEM), in which the entries indicate the bond order between connected atoms.^6^ This representation can be helpful to implement chemical reactions through a matrix addition operation of the reactants and products. Feng et al.^16^ combined two different representations of the molecules: (1) structural units, which represent the basic structural components of the molecules; and (2) a bond-electron matrix, which represents the atom connectivity of the molecules. Using a group contribution method, the framework can predict petroleum molecules’ physical and chemical properties based on their chemical structures. The method was validated for low-carbon number components, and good agreement was observed between the predicted and experimental properties. On the other hand, when working with high-molecular-weight molecules that contain a large number of atoms, such as asphaltenes and resins, especially in the case of mixtures of multiple heavy molecules as found in heavy fractions of petroleum, this approach requires manipulating numerous high-order matrices.

Yet another way to represent molecules is based on graph theory. At the most fundamental level, a molecule is defined as a combination of chemically bonded atoms arranged in a specific manner. This theory, as a fundamental branch of mathematics, can be used as a powerful tool for modeling and comprehending complex systems by representing object relationships as graphs.^17^ The molecule can be represented as a graph where the atoms are represented by individual vertices, and bonds connecting a pair of atoms are represented by edges that connect the respective pairs of vertices. Ring structures can also be represented by individual vertices in the graph.^18^

Representations based on this theory can provide detailed information about molecules and have been widely used for various purposes. The molecule can also be decomposed into fragments connected in a graph, where fingerprints generated by a pretrained encoder are integrated to create an invariant and accurate molecular representation.^19^ Molecular representation is essential for different methods of molecular manipulation, from classical approaches to those using machine learning, because it converts molecular structures into usable data, allowing models to make more accurate predictions.^20^ Particularly for calculating properties using group contribution methods, as it is possible to identify the arrangement of atoms that compose the groups by inspecting the graphs.^21,22^ However, when this property calculation is embedded in a computationally expensive algorithm (such as the molecular reconstructions and reaction models mentioned earlier), this inspection can significantly increase computational cost compared to more simplified approaches like SOL. Additionally, despite using the molecular graph in their methodologies, the authors do not explicitly explain how this representation occurs within their algorithms. Therefore, based on graph theory, we present a matrix for representing the molecule as a graph (MAG) listing the edges of the molecules, along with some additional information.

Each presented methodology has its own advantages and disadvantages, depending on the required application, but a representation that preserves the maximum amount of molecular information while maintaining the necessary simplicity in computational manipulation was pursued. Therefore, to deal with complex molecules in complex systems, this work proposes a molecular representation with the combination of two different matrices. The first of these is an extended version of Structure-Oriented-Lumping (SOLex), representing each core in a molecule with 44 increments organized into vectors, capable of describing multicores structures, and the second one is the Molecule as a Graph matrix (MAG), which describes a molecular graph providing detailed information about the bonds between atoms in a molecule. The combined use of these representations integrates the agility of SOL (allowing for quickly determining the existence of structures in the molecule) with the structural detail that a molecular graph representation like MAG possesses. This allows for a comprehensive detailing and easily related to thermodynamic properties and reactive manipulation of hydrocarbon molecules, even with the presence of heteroatoms. By combining these matrices, it becomes possible to achieve a high level of molecular detail and enables easy computational manipulation and straightforward visualization of the connections between atoms.

## 2 Methodology

The proposed molecular representation consists of two stages: attribute representation based on the extended version of SOL methodology (SOLex), and a bond matrix that allows identification of atoms and bond types connecting them in a molecule, defined as Molecule as a Graph matrix (MAG). Each part of the representation has characteristics that enable the detailed description of different aspects of the molecule. The combination of these characteristics favors the development of manipulating and analysis for each structure, as the calculation of properties and application of chemical reactions, provided that the appropriate kinetic route is known.

The separation of the representation into different matrices allows obtaining multiple levels of molecular detail. When the global information is required, such as the number of rings, the SOLex matrix can be used to obtain a quick response. On the other hand, when detailed information about the connections between atoms is necessary, the use of the Molecule as a Graph (MAG) matrix becomes essential. The combined use of these two representations enables obtaining the desired information about a molecule in a detailed and efficient manner. Utilizing the extended SOL matrix (SOLex) enables the investigation of particular attributes within the molecule. For instance, it facilitates the detection of distinct structures like heteroatoms and ring types. If the goal is to discern in which core this information resides, this matrix also provides core-specific insights. This, in turn, supports a more detailed examination of the distinct features present in each core. Only when identifying the exact position of the desired attribute is necessary does the MAG matrix come into play, offering an advanced level of molecular detailing. This involves precise atom localization, information regarding chemical bonds, structural classifications, and core organization. In essence, the integration of these two representations avoids unnecessary MAG matrix inspections, which, otherwise, would require increased computational resources due to the imperative need to analyze the entire molecular structure to identify each attribute.

Furthermore, the use of the two distinct matrices can be customized according to the specific application, providing both flexibility and precision. This makes the integration of these two representations highly compelling, ensuring, the freedom of choice throughout an application. An illustrative application scenario involves the detection of specific structures required for a reaction rule, using the SOLex matrix. Once a positive identification is achieved, the MAG matrix comes into play, providing detailed insights into the reaction by determining the atoms and bonds that are involved. Next, we will present our molecular representations (MAG-SOLex) and how to use them.

### 2.1 Extended SOL (SOLex): New Attributes for Structure-Oriented Lumping

Quann and Jaffe^7^ developed the SOL method, which mathematically organizes a set of increments into a vector. Each vector element corresponds to one of the increments, allowing a molecule to be represented as a numerical vector with 22 elements. The unique combination of numbers and types of increments distinguishes different molecules. A mixture of molecules is then represented by a matrix, where each row corresponds to one molecule. These structures were modified by Jaffe et al.^14^ to better represent the vacuum residue stream from oil mixture, allowing for the representation of multicore molecules and the inclusion of typical heteroatoms found in this kind of mixture, such as Nickel and Vanadium, resulting in a total of 24 columns.

**Figure 1:**
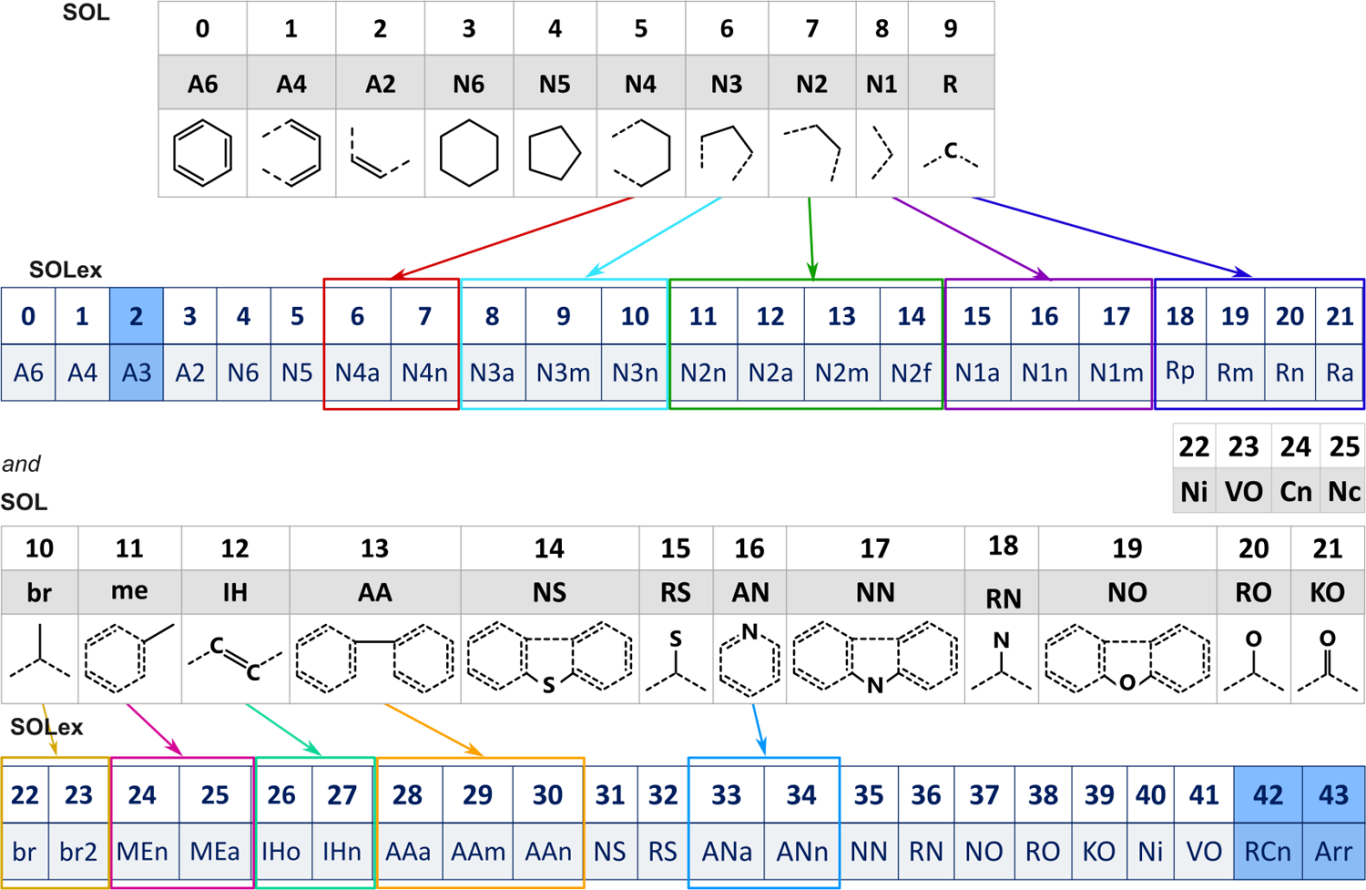
Extended SOL (SOLex): attributes adaptation.

Later, Maciel^11^ proposed an extended version of the SOL representation to provide a more detailed description of the structures, and also to easily obtain the groups of Joback and Reid^23^ from the extended SOL through a simple matrix multiplication. This will be discussed later in the property calculation section. For this, they needed to expand some SOL attributes into more detailed structures. For instance, **N2** rings must be connected to three other rings, which can be either aromatic (**A6**, **A4**) or naphthenic (**N6**, **N4**), or both. The presence of an **N2** group in the molecule leads to a different accounting of the Joback and Reid^23^ groups if this ring is connected to aromatic or naphthenic structures. Using the original SOL representation, it is not possible to differentiate these possibilities, which may result in an incorrect accounting of the groups by Joback and Reid.^23^ To address this issue, Maciel^11^ proposed an expansion of the **N2** group in SOLex, considering four possibilities: **N2n**, where the connected rings are all naphthenic; **N2a**, where the **N2** ring is connected to three other aromatic rings; **N2m**, where two connected rings are naphthenic and the other is aromatic; and **N2f**, which concatenates two aromatic rings and one naphthenic ring with the one being analyzed. The same logic was applied to the other expansions proposed by Maciel,^11^ as demonstrated in Figure 1 and Table 1.

**Table 1:**
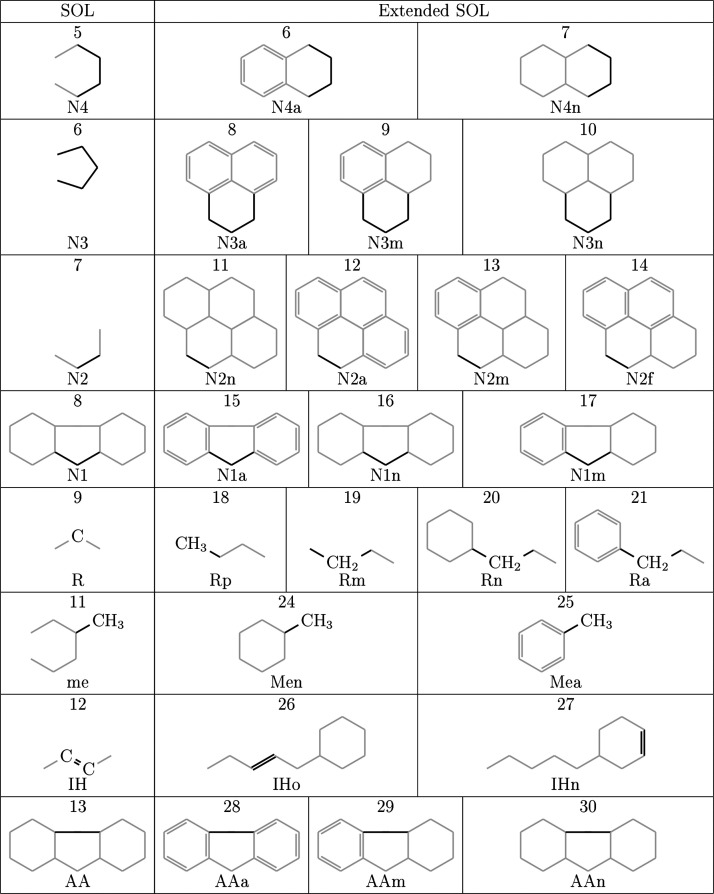
Extended SOL (SOLex): New Attributes Details

Based on the works of Jaffe et al.^14^ and Maciel,^11^ this work proposes a new extended version of the SOL representation, called SOLex, which provides a more detailed description of the structures contained in each core, without losing the global information about the entire molecule. To achieve this, the proposed SOLex matrix is now three-dimensional. In this representation, the first position of the dimension referring to the cores (position 0) presents the global values of the molecule (i.e., the sum of those attributes across all cores). This way, both global and per-core information are included in the same representation.

In addition to the expansions made by Maciel,^11^ the group **A3** was included to encompass aromatic structures, such as Phenalene. The **N3** group was expanded into **N3a**, **N3m**, and **N3n** to accommodate this structure connected to two aromatic rings, one aromatic and one naphthenic, and two naphthenic rings, respectively. Similarly, the group **br2** was included to account for quaternary carbons in aliphatic chains. Furthermore, to facilitate the management of information and the identification of core arrangements, two additional attributes have been added (**RCn** and **Arr**), totaling 44.

The **RCn** pertains to the connectivity between cores, detailing the number of aliphatic carbons connecting the cores. This attribute presents the value of the total number of aliphatic carbons connecting the cores in the global position of the molecule (position 0 of the core dimension), and it presents a code of 4 pairs of concatenated digits (totaling 8 digits **RCn** = WWXXYYZZ) referring to the sizes of the chains connecting the cores.

The **Arr** relates to the molecule’s arrangement, presenting the total number of cores and indicating which cores are connected. This attribute presents the total number of cores in the global position of the molecule (position 0 of the core dimension), and it presents a code of 4 digits concatenated (**Arr** = WXYZ) indicating to which other cores that core is connected. The contributions in the SOL expansion (expansion of the **N3** attribute and addition of the **A3**, **RCn** and **Arr** columns) are also presented in Table 1 and Figure 1. If one is working only with single-core molecules, there is no need to include the third dimension, nor the **RCn** and **Arr** columns, which makes the manipulation of SOLex even simpler. To illustrate the proposed SOLex representation, it will be applied to both single-core and multicore molecules as described below.

#### 2.1.1 Monocore Molecules

**Figure 2:**
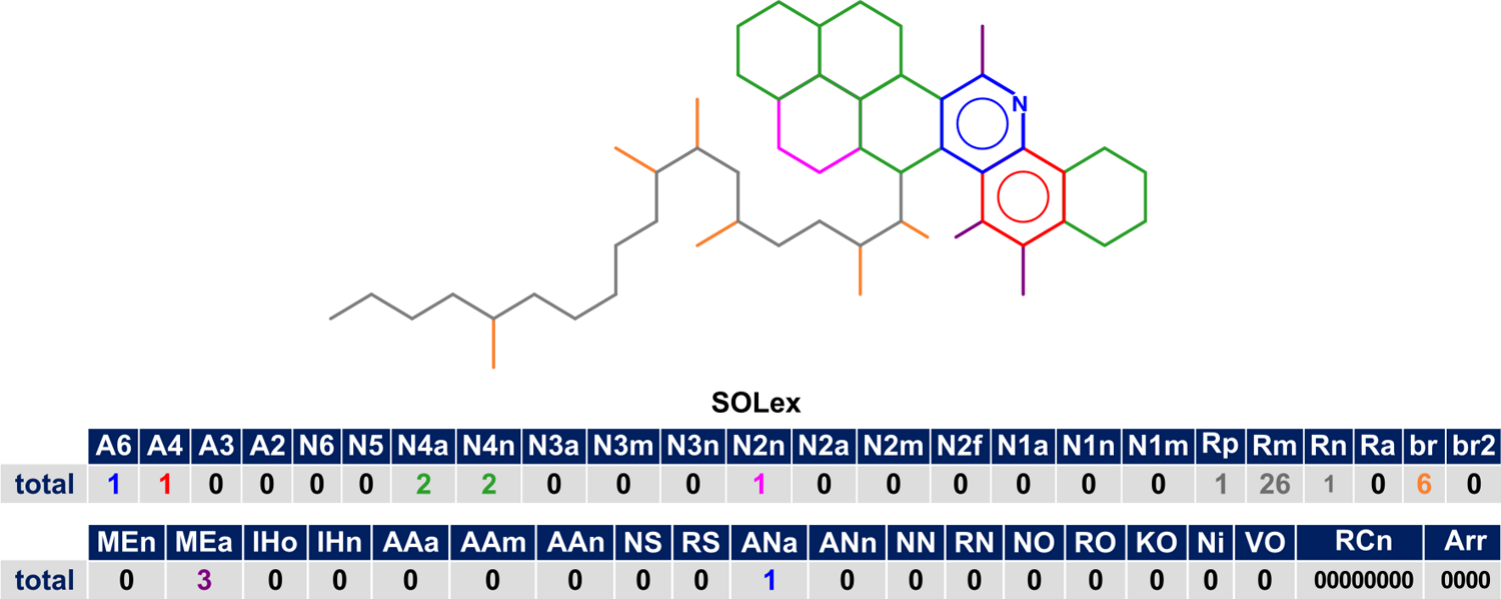
SOLex representation for a monocore molecule: total attributes for Example 1.

Simple molecules are considered those with only one ring group concatenated, known here as single-core or monocore molecules. The columns **RCn** (42) and **Arr** (43) represent the connectivity between cores and their arrangement, respectively, which do not provide additional information to the representation of a molecule when considering only one core. Thus, the information contained in the global position and in the position referring to the first (and only) core are the same. A color illustration of a monocore molecule with the SOLex matrix is shown in Figure 2, where different attributes are represented with various colors for a better understanding of the matrix. Naphthenic and aromatic rings increments are present, as well as 27 carbon atoms represented in columns **Rp** and **Rm**, which are distributed throughout the molecule between the branched aliphatic chain attached to the naphthenic ring and methyl groups. The **br**, **MEa** and **MEn** groups denote six branching points of the chain and three methyl groups, respectively.

#### 2.1.2 Multicore Molecules

Jaffe et al.^14^ represented multicore molecules as being composed of single-core ones. The molecules proposed and employed in this representation methodology were named according to groupings of rings within them, which are considered as cores and are convenient for representing very high molecular weights. This approach allows for representing asphaltenic molecules as explained by Mullins et al.,^24^ providing detailed explanations and illustrated examples.

**Figure 3:**
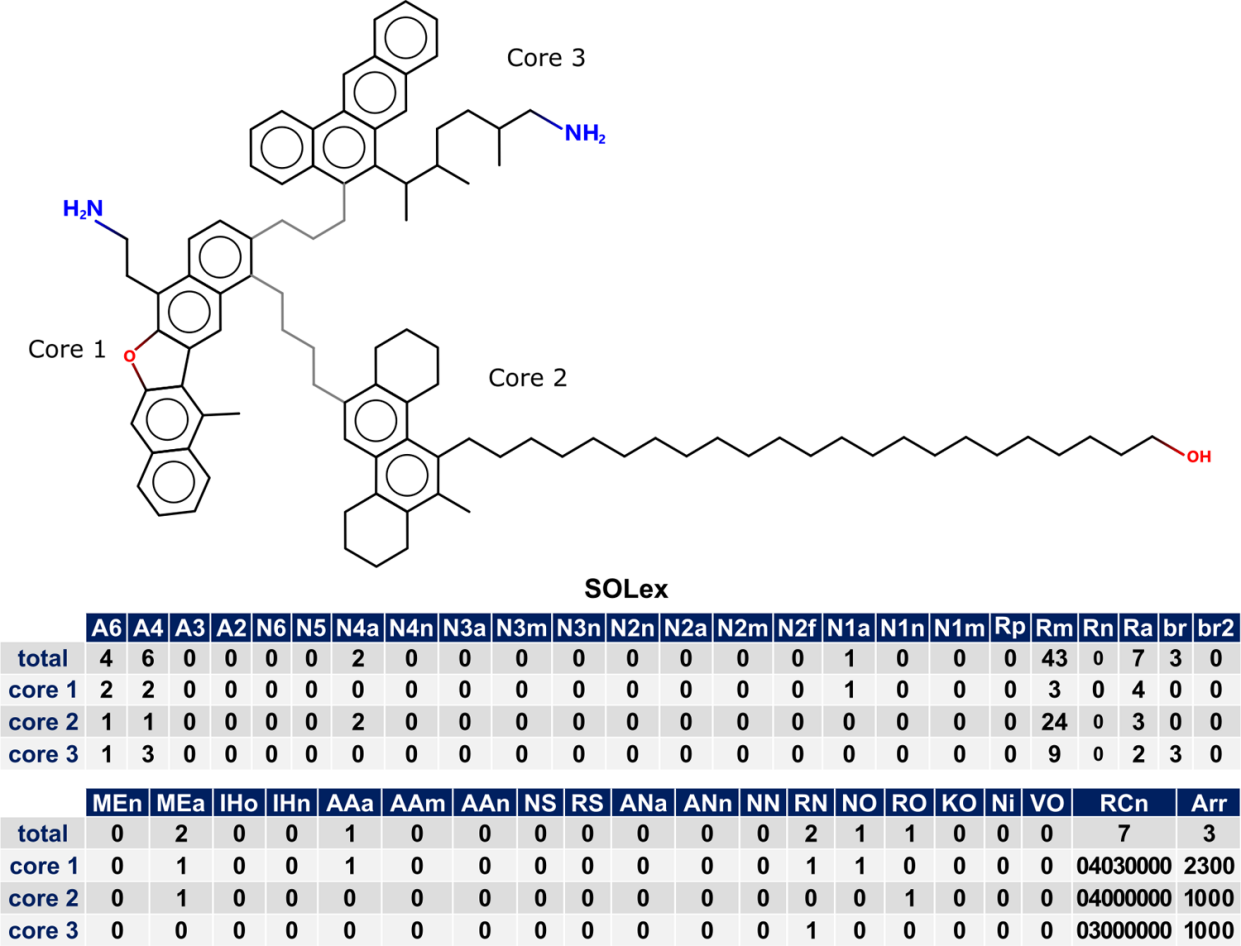
SOLex representation for a multicore molecule: total and cores attributes for Example 2.

The provided example, as illustrated in Figure 3, demonstrates the SOLex representation of a multicore molecule consisting of three cores, as indicated in the first row of the **Arr** column. The global values of the molecule are presented in the first row, which correspond to the sum of the attributes of each core. In the second row, representing core 1, there are 2 aromatic rings of type **A6**, 2 rings of type **A4**, and 3 linear carbons (**Rm** = 3), along with Nitrogen and Oxygen heteroatoms represented by **RN** and **NO**. Core 1 is connected to cores 2 and 3, as denoted in the **RCn** column (2300), with four atoms between cores 1 and 2, and three atoms between cores 1 and 3 (**RCn** = 0403YYZZ). The third row, representing core 2, contains the rings, heteroatoms, and 24 linear carbon atoms (**Rm** = 24) distributed throughout this region of the molecule. The bond with core 1 is also represented in the **RCn** column of core 2 as 04XXYYZZ. The fourth row, representing core 3, consists of a set of rings with a side branched chain with 9 carbons (**Rm** = 9), connected to core 1 by three atoms (**RCn** = 03XXYYZZ). As the molecule only has three cores, the remaining digits of the attributes **RCn** and **Arr** are set to zero. Note that the first row of the **RCn** column presents the total number of aliphatic carbons connecting the cores (**RCn**[0] = 3 + 4 = 7), and that the global value of aliphatic carbons in the molecule includes those used to connect the cores (**Rm**[0] = 3 + 24 + 9 + **7** = 43). Additional attributes can be found in the other columns.

It is possible to obtain a matrix representing the number of atoms in each molecule from the SOLex representation. To do this, multiply the zero position of the core dimension of the mixture’s SOLex by the stoichiometric matrix, adapted from,^7^ as presented in the Supporting Information. The result of this multiplication is a matrix containing the quantities of C, H, S, N, O, Ni, and V atoms in each molecule.

The SOLex is our first and simplest proposed representation. It allows for detailing the types of carbons present in the molecule, but it is not possible to map the entire molecule, identify how the atoms are bonded or differentiate isomers. Therefore, for a more detailed identification of the structures contained in the molecules, we propose a second representation of the molecule as a graph in the next section.

### 2.2 MAG Matrix: Molecule as a Graph Representation

The proposed molecular representation as a graph (MAG) aims to provide a detailed description of the structure of molecules by identifying the types of atoms present, their respective connections, and the bond types between them, as well as other relevant information. It was initially designed to represent hydrocarbons from petroleum and biomass-derived sources, but it can be easily adapted to represent any type of molecule.

The structural arrangement of this representation differs significantly from previous methodologies. We propose a single matrix to represent each molecule as a graph. For this, each atom is numbered (starting from zero), with the exception of hydrogen, which is implicitly included to complete the valence of the represented elements. Additionally, the bonds, rings, and cores must also be numbered (starting from 0, except for the cores, which start at 1).

**Figure 4:**
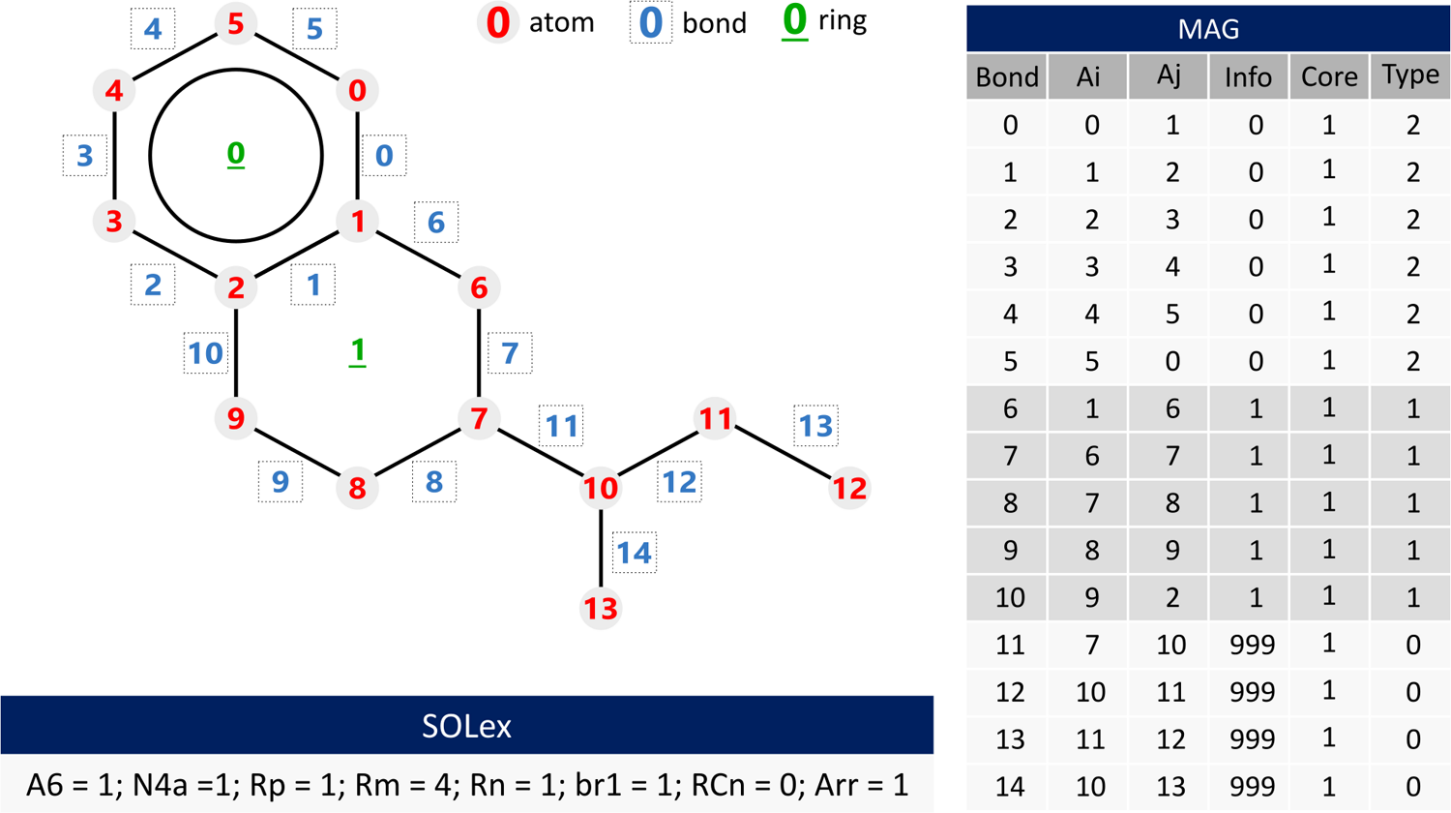
Molecule example 3: SOLex and MAG representation.

It is recommended that atom and bond numbering not be random and that it follows a coherent order based on the connections between atoms in the molecules. Numbering should start within each nucleus, with atoms belonging to aromatic rings, followed by atoms in naphthenic rings, methyl connected to rings, the main aliphatic chain, branches of the aliphatic chain, and heteroatoms of the aliphatic chain, as observed in Figure 4. The order of atom numbering does not affect the computational functionalities of the MAG, but it facilitates the interpretation of its content.

Each row of the MAG matrix represents one edge of the graph, which is a connection between two previously numbered atoms. The matrix comprises five columns, as summarized in Table 2: the first column denotes the number of the first atom involved in the bond (**Ai**); the second column denotes the number of the second atom (**Aj**); the third column provides complementary information about the bond (**Info**), which may include the number of the ring to which the bond belongs (if applicable), codes for heteroatoms (1000-S, 1001-N, 1002-OH, 1003-O and 1004-OOH) if it is in the second position (**Aj**), the code 999 if it is between aliphatic carbons, or the codes 900 and 901 when it is necessary to identify *cis* and *trans* configurations, respectively; the fourth column denotes the core to which the bond belongs (**Core**), or the code 998 if it belongs to some carbon chain that connects cores; and the fifth column denotes the type of bond (**Type**), which can be 0 for saturated aliphatic, 1 for saturated naphthenic, 2 for unsaturated aromatic, 3 for unsaturated aliphatic, 4 for unsaturated naphthenic, 5 for a bond between a metal and N (typical of porphyrins), and 6 for triple bonds.

**Table 2:** MAG Matrix Columns Details: Ai, Aj, Info, Core, and Type

MAG matrix details		
**Ai**	first atom in the bond	
**Aj**	second atom in the bond	
**Info**	ring number	
	S heteroatom	1000
	N heteroatom	1001
	OH heteroatom	1002
	O heteroatom	1003
	OOH heteroatom (carboxyl)	1004
	aliphatic carbon	999
	*cis* configuration	900
	*trans* configuration	901
**Core**	core number	
	carbon chain that connect cores	998
**Type**	saturated aliphatic	0
	saturated naphthenic	1
	unsaturated aromatic	2
	unsaturated aliphatic	3
	unsaturated naphthenic	4
	bond between a metal and N	5
	triple bonds	6

As can be seen in Table 2, it is possible and simple to add new atoms to the Info column and include different specifications related to the type of bonds. This feature enables the description of several mixtures without requiring a significant implementation effort. Furthermore, the MAG matrix is adaptable for drawing molecules and can be easily translated into SMILES^2^ format.

An example molecule is illustrated in Figure 4 to demonstrate the application of the MAG and SOLex representations. The ring 0 (**Info** = 0) has bonds 0 to 5, which are type 2, i.e., unsaturated aromatic bonds. Since the molecule has a simple monocore structure, the Core column has only one value (1). The ring 1 (**Info** = 1), on the other hand, is a saturated structure (**Type** = 1) that adds five bonds to the matrix (6–10), connecting atoms 6–9 to atoms 1 and 2 of the previous ring, respectively. The branched aliphatic chain is represented by bonds 11–14.

The MAG matrix for the monocore molecule, Example 1, illustrated previously in Figure 2, is presented in Supporting Information and shows the 61 bonds between the atoms, including rings, nitrogen atom, methyl groups, and the aliphatic chain. The second example of a Multicore molecule (Figure 3) is illustrated in the same section and it is possible to verify the connections of each core as well as the chain linking them.

**Figure 5:**
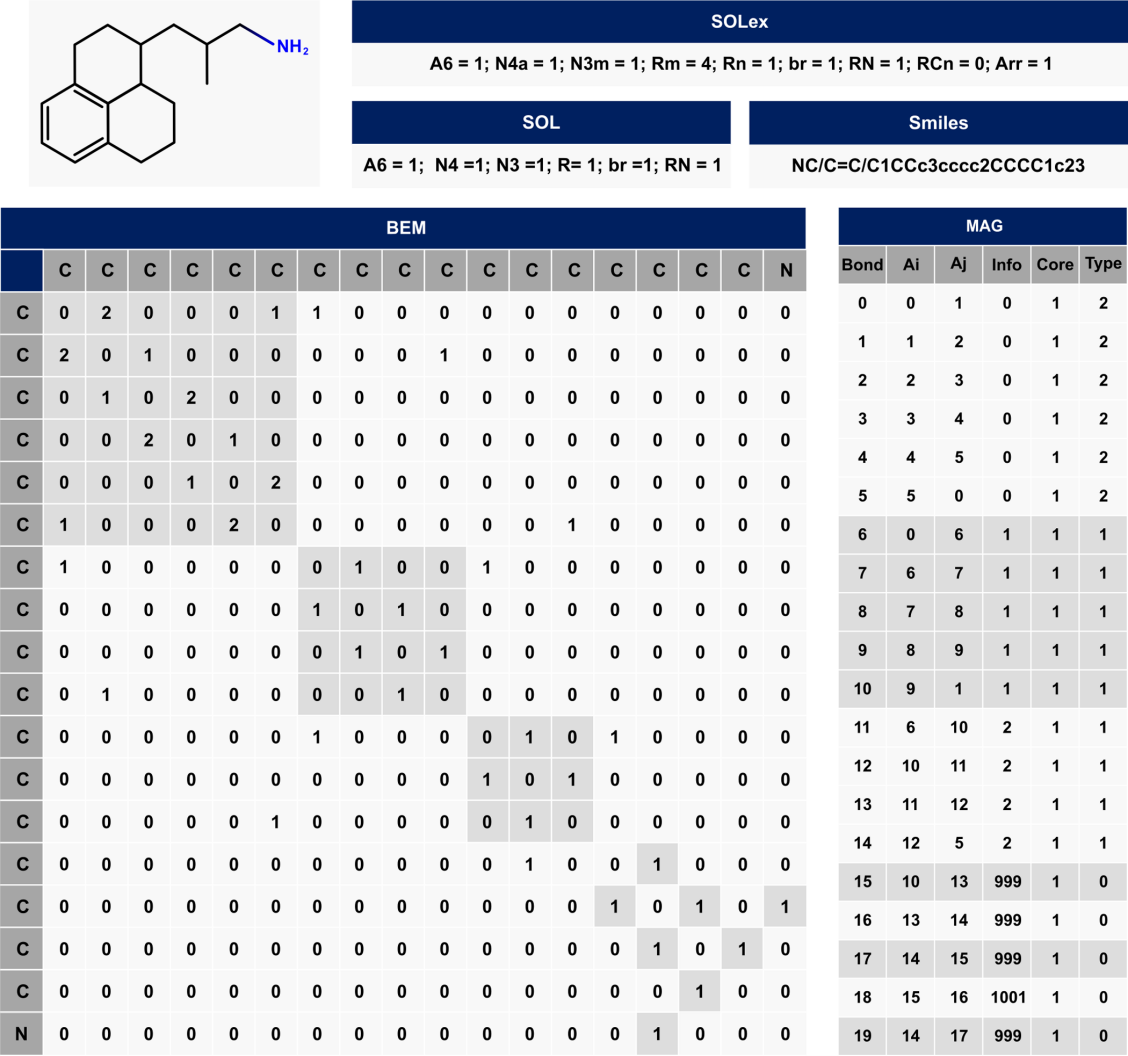
Molecule example 4: Smiles, BEM, SOL, SOLex and MAG representation.

For visualization and comparison purposes, the Figure 5 showcases various approaches to represent a hydrocarbon molecule with an NH_2_ heteroatom: SMILES (Bjerrum^2^), connectivity matrix (BEM) (Klein et al.^6^), SOL (Quann and Jaffe^7^), SOLex, and MAG representation. The detailed information in the SOLex matrix allows the identification of the **N4** ring type, which is an **N4a** due to its connection to an unsaturated ring (**A6**). Additionally, there is a linear chain connected to a naphthenic ring (**Rn**). The MAG matrix identifies 20 atom bonds: 0–5 belonging to ring 0 (unsaturated), 6–10 to ring 1 (saturated), and 11–14 to ring 2 (saturated). Bond 18, with “1001” in the Info Column, represents the Nitrogen atom at **Aj** position. Furthermore, the image includes the connectivity matrix (BEM) and the SMILES representation of the molecule. It is important to notice that the MAG matrix increases by 1 row for each connection added, but the number of columns remains fixed, unlike the BEM matrix, which increases in both directions when considering an additional atom in the molecule.

The two proposed molecular representations presented in this work are independent and can be used separately or together. When only simpler molecular information is required, it is convenient to use SOLex. When more detailed molecular information is required in a simple algorithm, MAG becomes the most suitable representation. When more detailed molecular information is required in a computationally expensive algorithm, the combined use of both representations is indicated, as will be presented in the examples in the next section.

## 3 Results and Discussion

### 3.1 Property Calculations Using Group Contribution Methods

#### 3.1.1 Joback and Reid’s Group Contribution Method

One of the simplest group contribution method for calculating thermodynamic properties is the Joback and Reid.^23^ It is possible to obtain a matrix of Joback groups using only the SOLex representation, which was one of Maciel’s^11^ original objectives when expanding SOL. This is achieved through matrix multiplication between the Joback Table, that can be found at the Supporting Information material and zero position of the core dimension of SOLex of the mixture, excluding the **RCn** and **Arr** columns. The Joback Table presents the relationship between the SOLex attributes and the 23 Joback groups that Maciel^11^ used to represent the molecules in his molecular reconstruction problem. This matrix multiplication results in a matrix with molecules in the rows and Joback groups in the columns, thereby enabling the calculation of properties of pure components such as critical temperature, critical pressure, acentric factor, ideal-gas heat capacity, liquid viscosity, boiling point, melting point, enthalpy of vaporization, enthalpy of formation, and Gibbs free energy of formation.

Due to its simplicity, it is interesting to obtain the Joback groups for a mixture, even though this group contribution method is not used for estimating thermodynamic properties. This is because it allows us to easily understand the types of carbons involved in the molecules. For example, it is possible to easily calculate the amount of saturated and unsaturated carbon, or the amount of naphthenic, aromatic, and olefinic carbon (analogous to results from nuclear magnetic resonance).

**Figure 6:**
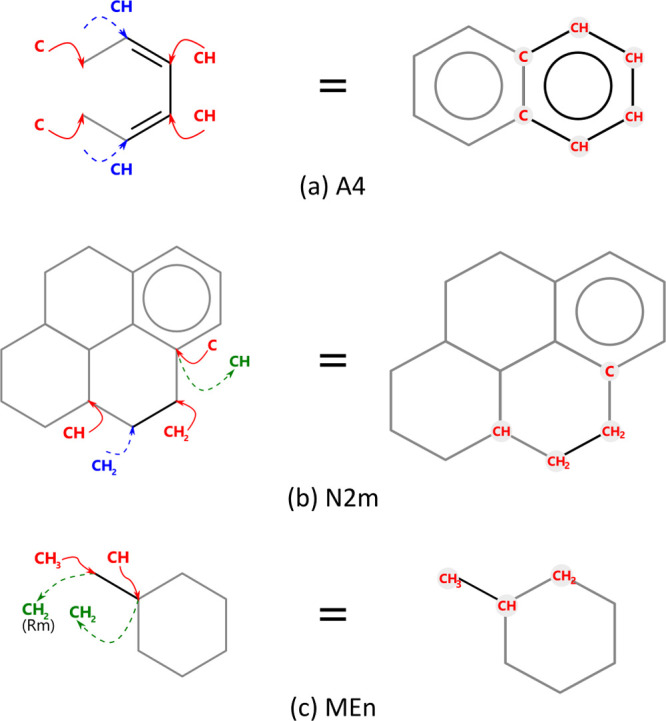
Example of accounting for Joback and Reid^23^ groups from the Extended SOL attributes implicit in the coefficients of the matrix presented in Joback Table: (a) **A4** attribute; (b) **N2m** attribute; (c) **MEn** attribute.

**Figure 7:**
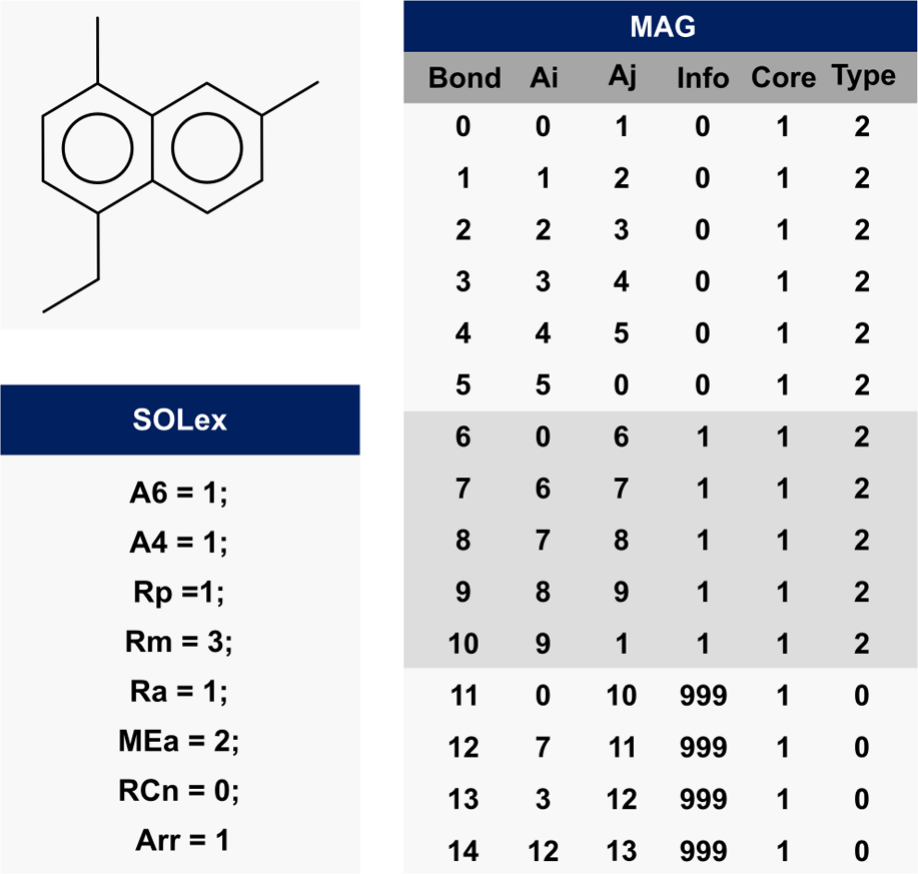
Representations MAG and SOLex for 4,6-dimethyl-1-ethylnaphthalene.

Analyzing the information contained in this table, it can be observed that certain attributes of the SOLex add atoms to the molecules. For instance, structures **A6**, **N6**, **N5**, **Rp**, and **Rm** exclusively possess positive values, resulting in an overall positive balance. Other attributes add atoms and modify existing ones, such as structures **N1**, **N2**, **N4**, and **A4**, which possess both positive and negative values, leading to a positive overall balance. Lastly, there are attributes that only modify existing atoms without adding new ones to the molecule. These attributes include structures **Rn**, **Ra**, **br**, **br2**, **MEa**, **MEn**, **IHo**, **IHn**, **AAa**, **AAn**, **NS**, **ANa**, **ANn**, **NN**, **NO**, and **KO**. When utilizing both positive and negative values, these attributes exhibit a overall balance equal to zero. Figure 6 provides a more detailed illustration of this concept, when adding an **A4**, two CH groups and two C groups are added to the molecule (red), as the previous ring already contains two CH groups (blue). With **MEn** and **N2m** attributes, besides adding and modifying structures of the molecules, CH and CH_2_ groups are removed from the counting (green), respectively, to maintain the correct global number of atoms. To illustrate the acquisition of the Joback groups, the matrix multiplication proposed between Joback Table and the SOLex matrix of the molecule 4,6-dimethyl-1-ethylnaphthalene, depicted in Figure 7 was applied. This operation yielded the matrix presented in Table 3. Using the contribution constants of these groups,^23^ it is possible to calculate the thermodynamic properties of this molecule, as presented in Table 5.

**Table 3:** Joback Groups Matrix for the Molecule from Figure 7

–CH_3_*nr*	–CH_2_–*nr*	aCH*r*	aC*r*_1_	aC-*r*_3_
aliphatic CH_3_	aliphatic CH_2_	aromatic CH	peripheral condensed aromatic	substituted aromatic
3	1	5	2	3

Although this group contribution method is simple and useful, it may be limited when used on high molecular weight molecules, such as those present in the heavier fractions of petroleum. For these cases, the next section presents how to use the combined SOLex and MAG approaches to obtain thermodynamic properties using more robust contribution methods, such as that of Marrero and Gani.^15^

#### 3.1.2 Marrero and Gani’s Group Contribution Method

When dealing with high molecular weight molecules, the Joback method exhibits low precision.^25,26^ In such cases, the use of the Marrero and Gani method (M&G) can be applied, as it takes into account molecular arrangement, interactions between functional groups, as well as other second and third-order contributions, in its property calculations. If computational effort is not a problem, as in the calculation of properties for just one molecule, only the MAG representation can be used. However, if this property calculation is part of a complex algorithm, the computational cost of inspecting the MAG (or any other detailed representation) of each molecule to find each M&G group can be problematic. In this context, the combined use of the two representations (MAG-SOLex) can significantly save computational effort. The structures are preidentified in SOLex, and only if a more detailed inspection of the molecule is necessary, the MAG is consulted. For instance, in molecular reconstruction problems, thousands of mixtures consisting of thousands of molecules each are generated, and the properties of each must be calculated. Therefore, the application of a fast and accurate property calculation is necessary.

The algorithm developed and applied in this work was written in C++, but it can be implemented in other languages. For this, an algorithm that uses both representations to identify the M&G groups can be applied, as presented in Algorithm 1. In this algorithm, the variable SOLex[*m*][*c*][*a*], consisting of 3 dimensions, represents the expanded SOL matrix, where the index *m* represents a molecule in the mixture, *c* represents the core (or the total quantities of the molecule at position 0), and *a* represents the SOLex attribute as previously presented in Figure 1. The algorithm implemented in C++ with the examples presented in the Results and Discussion section are available on Github, as presented in Associated Content.
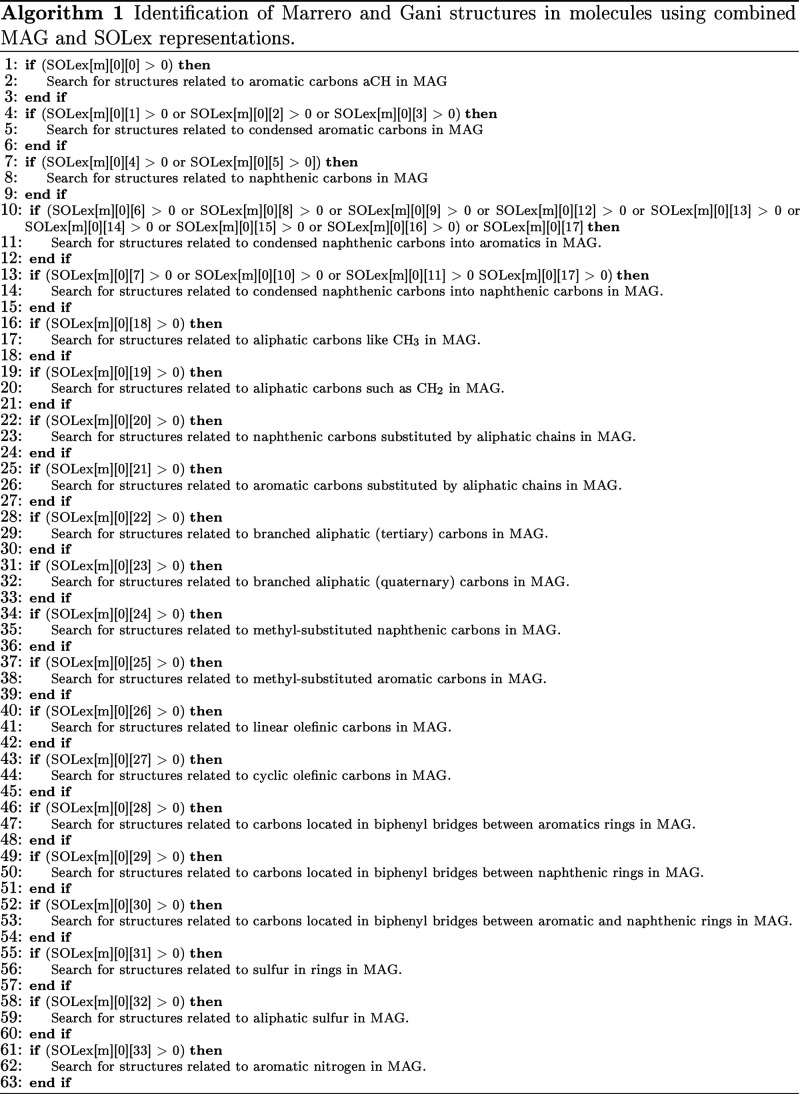

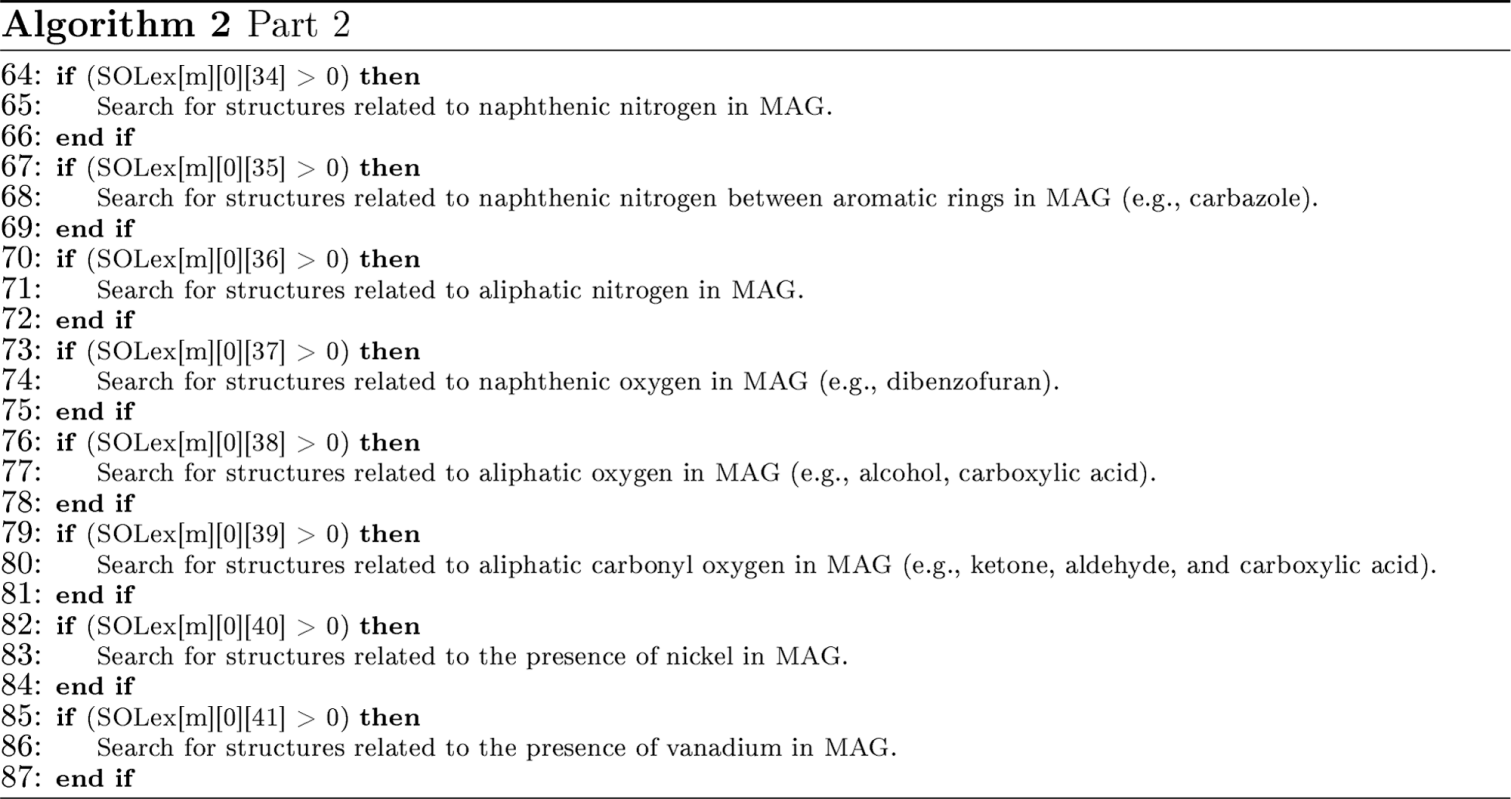


As seen in Algorithm 1, this structure of **if** and **else** statements avoids unnecessary inspections in the MAG. If the recommendations for atom and bond numbering, as well as the overall ordering of MAG, have been followed, the nucleus-specific information contained in the SOLex can be used to guide the region of the MAG to be inspected, making MAG readings even more objective and specific. This symbiosis between the two representations (MAG and SOLex) is the key to efficiently handling complex molecules in complex algorithms.

To exemplify the computational effort savings achieved with the application of this algorithm, thermodynamic properties were calculated using the Marrero and Gani group contribution method for a mixture of 5000 molecules in two situations. In the first one, only the MAG was used, where this matrix was exhaustively scanned to find each M&G group, resulting in a time of **7s**. In the second situation, the MAG and SOLex were combined, as proposed by Algorithm 1, resulting in a time of **3s**. The methodology was applied using a workstation equipped with an Intel Xeon 6244 processor, featuring 32 processing cores with a maximum clock speed of 4.4 GHz, running on the CentOS 7.0 operating system. These were the computational times for calculating properties of just one mixture. In molecular reconstruction problems, it is common to generate thousands of mixtures of 5000 molecules, and the difference in time spent for property calculation becomes significant. As can be observed, the difference in computational time in this example highlights the efficiency gain of using both representations combined in complex algorithms.

To illustrate the M&G groups obtained in a molecule, this strategy was used to find these groups for the molecule presented in Figure 7. The M&G groups found in this molecule are presented in Table 4, and the properties obtained from these groups in Table 5.

**Table 4:** Marrero and Gani^15^ First Order Groups Matrix for the Molecule from Figure 7

M&G first order group	amount
–CH_3_	1
aCH	5
aCfaC	2
aC–CH_3_	2
aC–CH_2_	1

**Table 5:** Comparison among the Experimental and Calculated Properties Using the Joback and Reid^23^ and M&G^15^ Group Contribution Methods for the Molecule Depicted in Figure 7

	exp	JR	M&G	JR % error	M&G % error
*T*_C_ [K]	811.44	773.68	804.21	4.7	0.9
*P*_C_ [bar]	24.24	32.06	24.58	–32.3	–1.4
*V*_c_ [cm^3^/mol]	633.5	608.5	591.2	3.9	6.7
*T*_b_ [K]	585.41	532.07	574.90	9.1	1.8
*T*_m_ [K]	333.04	459.87	333.8	–38.1	–0.2

As observed from the percentage errors, the Marrero and Gani method (considering only the first order) provided better property estimations than the Joback method, except for the Critical Volume. Additionally, the prediction errors of the Joback method tend to increase with the molecular weight of the molecules.^25,26^

**Figure 8:**
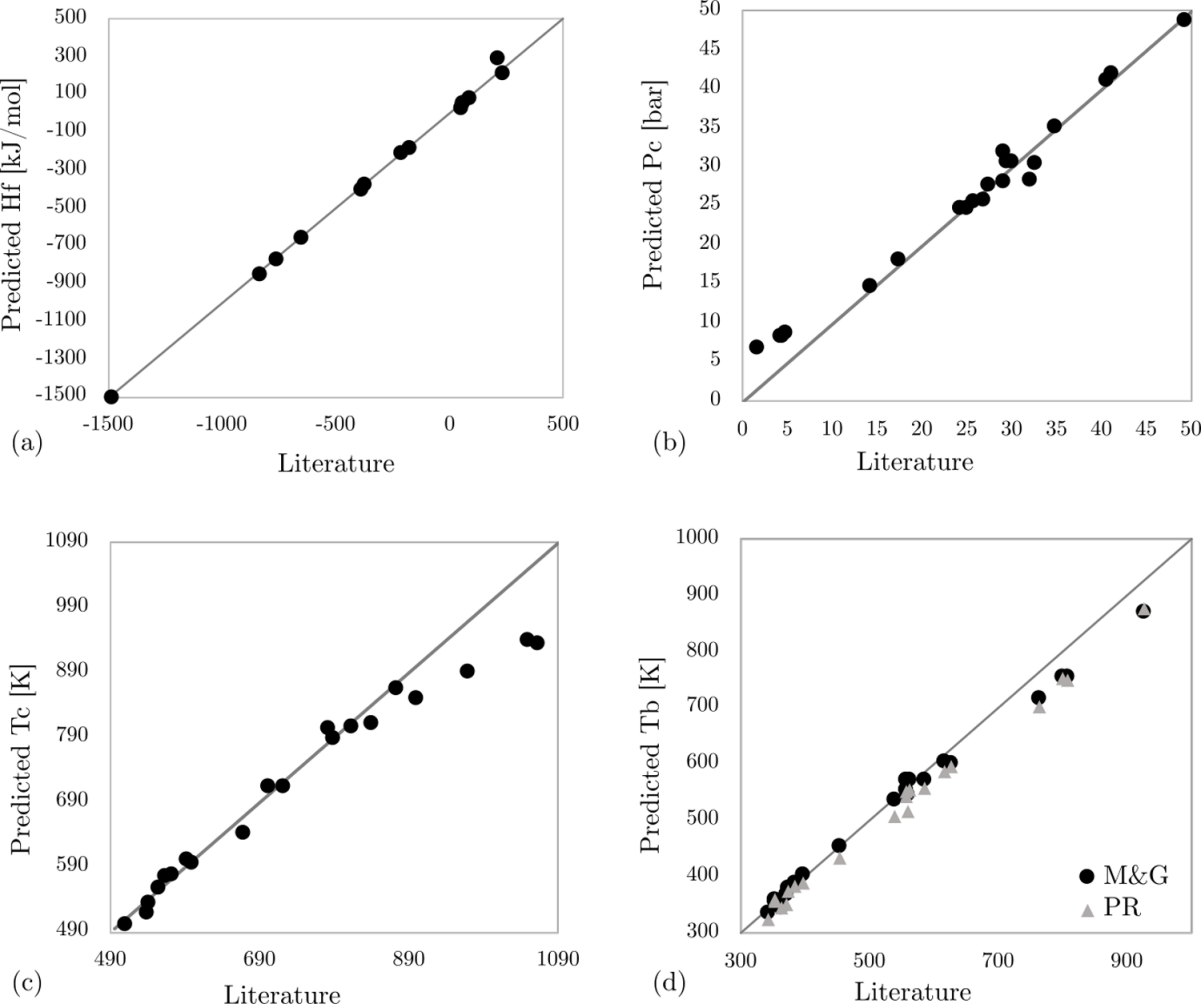
Comparison between calculated by the group contribution methods and experimental values for the following properties Yaws:^27^ (a) enthalpy of formation by M&G, (b) critical pressure by M&G, (c) critical temperature by M&G, and (d) normal boiling temperature by M&G and Peng–Robinson (PR). Calculated boiling temperature using the Peng–Robinson EoS was performed using the critical temperatures and pressures estimated by M&G group contribution and the acentric factors calculated using the group contribution method of Tahami et al.^28^ methods.

To test the accuracy of property predictions overall using the first-order M&G method, the methodology proposed in Algorithm 1 was used to identify and calculate the properties for 20 molecules with common organic functions and high molecular weight. These results were compared with the information available in Yaws,^27^ as shown in Figure 8, indicating that the selected models accurately predicted the properties.

As observed, through the combined use of both representations, it is possible to obtain the thermodynamic properties of complex mixtures (even with a large number of molecules) accurately and efficiently. Next, a demonstration of how to manipulate these representations to carry out chemical reactions will be presented.

### 3.2 Chemical Reactions

The proposed representation in reaction networks is demonstrated using the molecule shown in Figure 4, which undergoes two reactions in no specific order and can occur individually.

1. Aromatic ring saturation: the **A6**-type ring present in the molecule, in the presence of hydrogen molecules (H_2_), becomes a naphthenic ring (**N6**).
2. Dealkylation: the chain attached to the naphthenic ring is dealkylated to form a linear paraffin, and the presence of hydrogen (H_2_) is also required, resulting in a methyl group attached to the ring.

**Figure 9:**
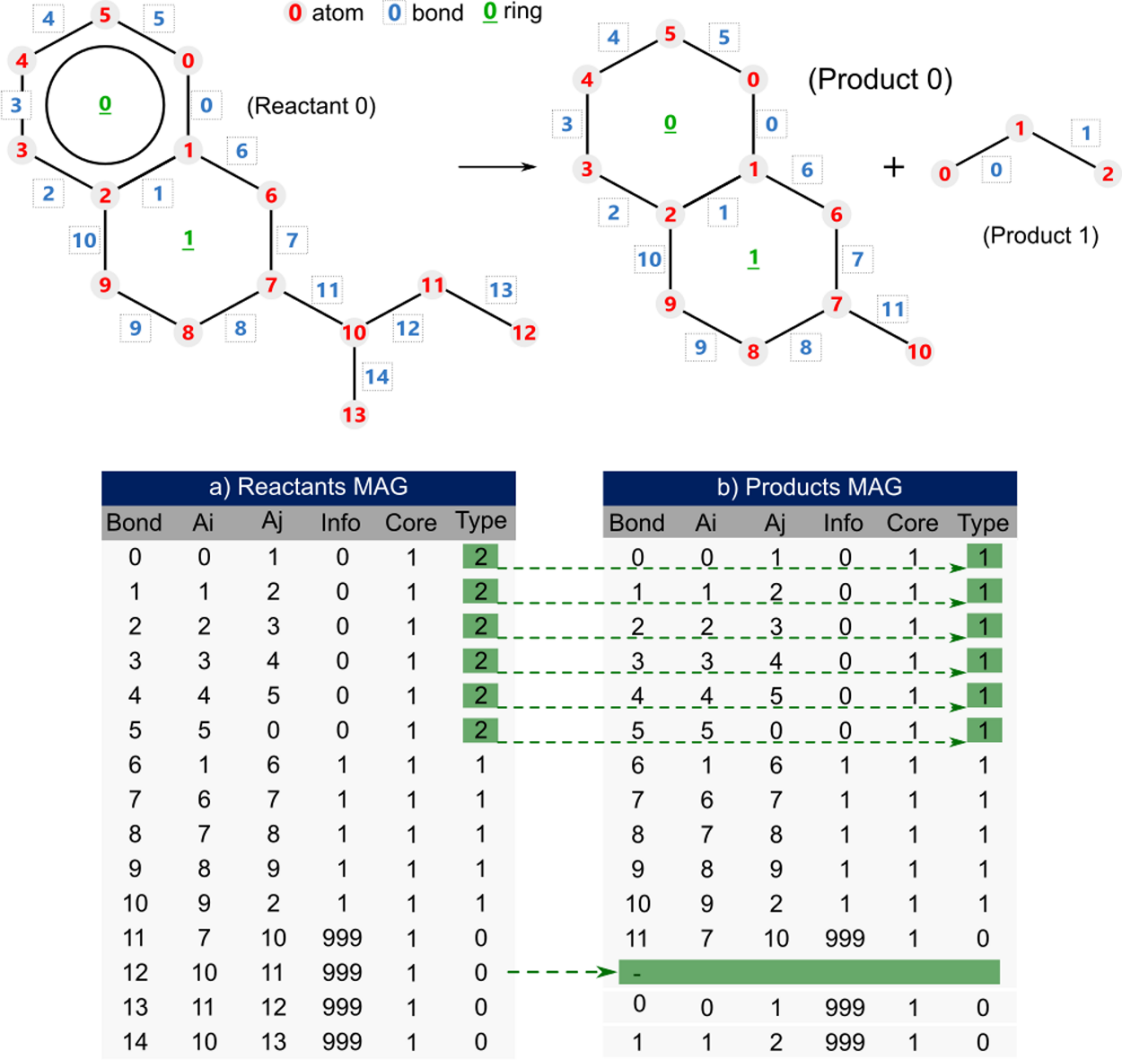
Reactions example: illustration and MAG Matrix for reactants and products.

The two products generated by these reactions and their MAG matrices are illustrated in Figure 9. The bond break resulting from the dealkylation reaction step is represented in the product matrix by the removal of the corresponding row (12); however, the number of atoms corresponds to the global balance. The aromatic ring saturation reaction can be observed by the change in the type of ring 0, from aromatic (**Type** = 2) to naphthenic (**Type** = 1), in rows 0 to 5.

**Figure 10:**
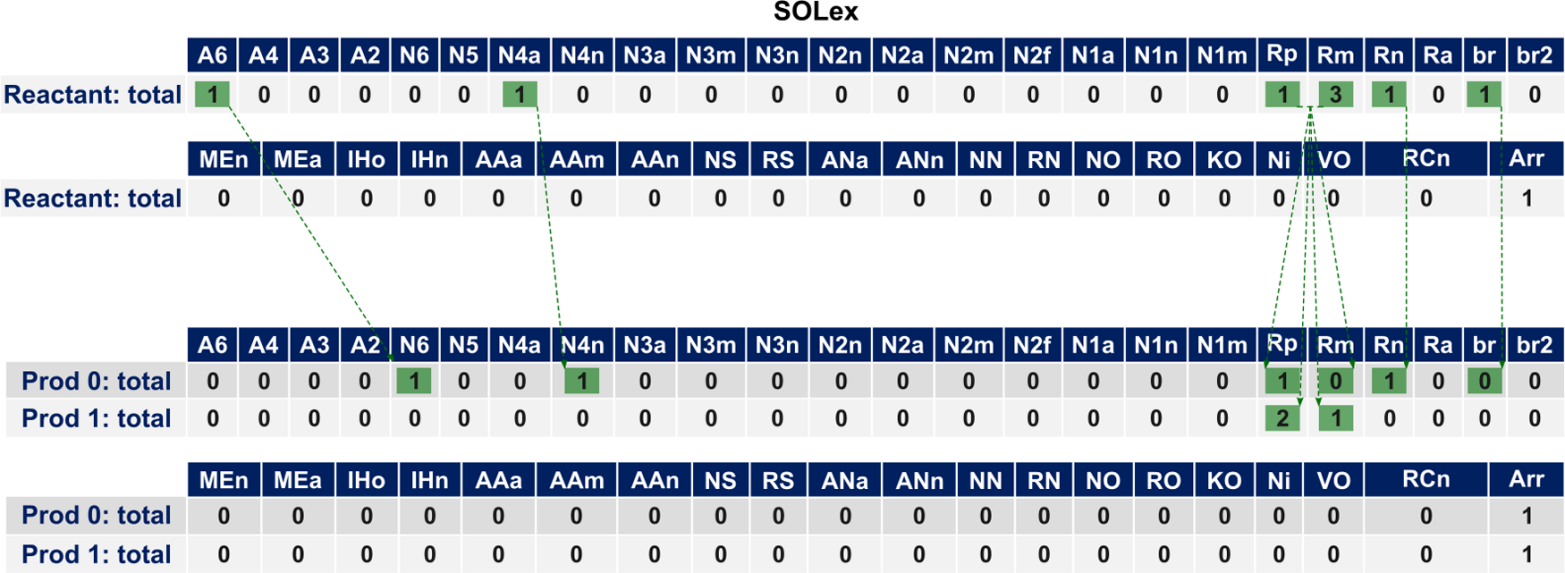
SOLex total line representation for the products of two reactions applied to the previous shown hydrocarbon.

The Figure 10 presents the transformations in the SOLex matrix for the same reactions applied to the molecule depicted in Figure 9. The saturation reaction of the aromatic ring can be observed by the transformation of the **A6** ring in the reactant to **N6** in the first product. Additionally, the naphthenic ring, which is conjugated to an aromatic ring (**N4a**) in the reactant, becomes conjugated to a naphthenic ring (**N4n**) in the first product. Similarly, the dealkylation reaction can be observed by the distribution of the alkyl carbons (present in the **Rp** and **Rm** columns) from the reactant among the products.

When it comes to reaction network algorithms, it is necessary to identify if molecules are subject to the available reactions. In this case, the combined use of the two representations (MAG-SOLex), once again, stands out in improving the performance of the algorithm. For this, it is possible to use a logic similar to Algorithm 1, where queries in SOLex are used to identify if the necessary structures for the reactions to occur are present in the molecules. For example, only if there are aromatic rings in the molecule (SOLex[*m*][0][0] + SOLex[*m*][0][1] + SOLex[*m*][0][2] > 0), it is subject to a aromatic ring saturation reaction. Similarly, only if there are alkyl chains present ((SOLex[*m*][0][19] + SOLex[*m*][0][20]) > 0), the molecule is subject to dealkylation.

The process of performing reactions using the proposed representations has been demonstrated, along with the utilization of combined representations to reduce computational effort in complex reaction algorithms. The following section will describe how to differentiate isomers using the MAG-SOLex representation.

### 3.3 Isomers Differentiation

Isomers are chemical compounds that share the same molecular formula, meaning they are composed of the same atoms but arranged in distinct configurations. This variation in molecular structure can result in significantly different physical and chemical properties among isomers. These disparities can directly impact reactivity, solubility, melting points, boiling points, and other characteristics of the compounds. Accurate identification of isomers is crucial for a precise understanding of compound properties and their appropriate application across various fields.

The SOLex representation was not designed to differentiate similar molecules like isomers. In this context, the use of MAG becomes essential. Once again, if computational efficiency is not important, only MAG can be used to differentiate molecules. However, the combined use of the representations can increase computational efficiency since it is only worthwhile to inspect the MAG to differentiate molecules if they are completely equal in the SOLex representation.

The heightened level of detail in the MAG matrix allows a more accurate representation of the molecular structure, thus significantly assisting in overcoming the challenges associated with isomer differentiation. Even cases of stereoisomerism can be differentiated, which is an interesting feature for a representation that does not have spatial coordinates. For example, geometric isomers can be distinguished due to the possibility of representing *cis* and *trans* structures (**Info** = 900 and 901, respectively). Similarly, optical isomers can be differentiated by identifying the order of bonds made by a quaternary carbon.

**Figure 11:**
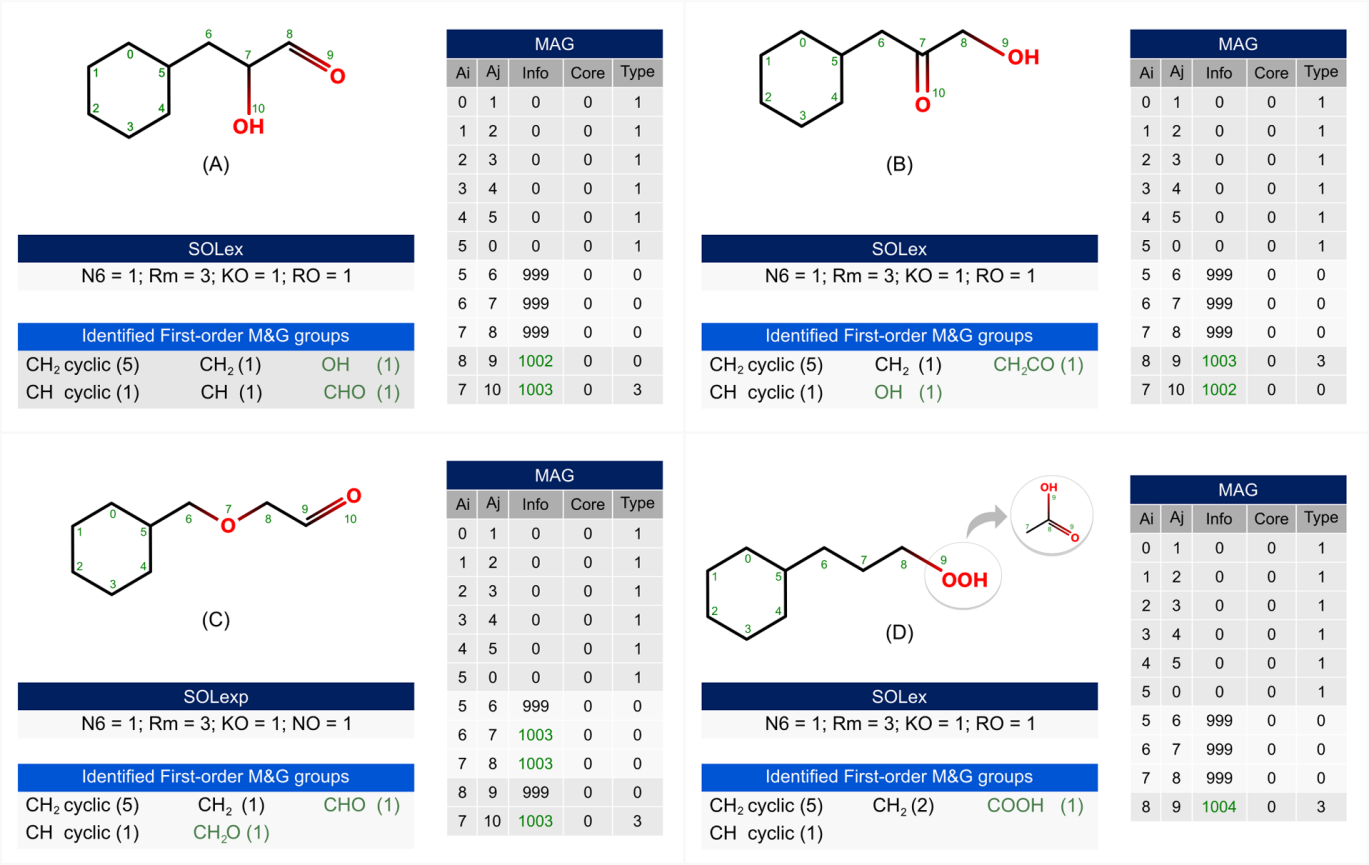
MAG and SOLex representations for isomeric oxygenated molecules with a naphtenic ring (N6) attached to a chain with (A) a ketone and an alcohol, (B) an aldehyde and an alcohol, (C) an ether and an aldehyde and, (D) a carboxylic acid.

To illustrate the differentiation of functional isomers, Figure 11 presents the representation of the two matrices for isomeric oxygenated molecules, including distinct functional groups attached to a naphthenic ring (**N6**). The structures represent: a ketone and an alcohol in case A, an aldehyde and an alcohol in case B, an ether and an aldehyde in case C, and a carboxylic acid in case D. The MAG matrix provides detailed information about each bond, enabling the identification of heteroatoms through the proposed encoding (1002 – OH, 1003 – O, and 1004 – OOH), as well as the atoms involved in their respective bonds. The first-order M&G groups identified for each molecule using MAG matrix are different. These groups are specified in Figure 11 and can be employed to calculate properties and to identify special compounds in the represented feeds.

This is an important differentiation of isomers for the petroleum industry. Naphthenic acids case D in Figure 11) are a group of acidic organic compounds that naturally occur in crude oil and can have various undesirable effects when present, such as corrosion and acidification of petroleum. The differentiation among oxygenated isomers is also important for the representation and differentiation of biomass-derived molecules.

**Figure 12:**
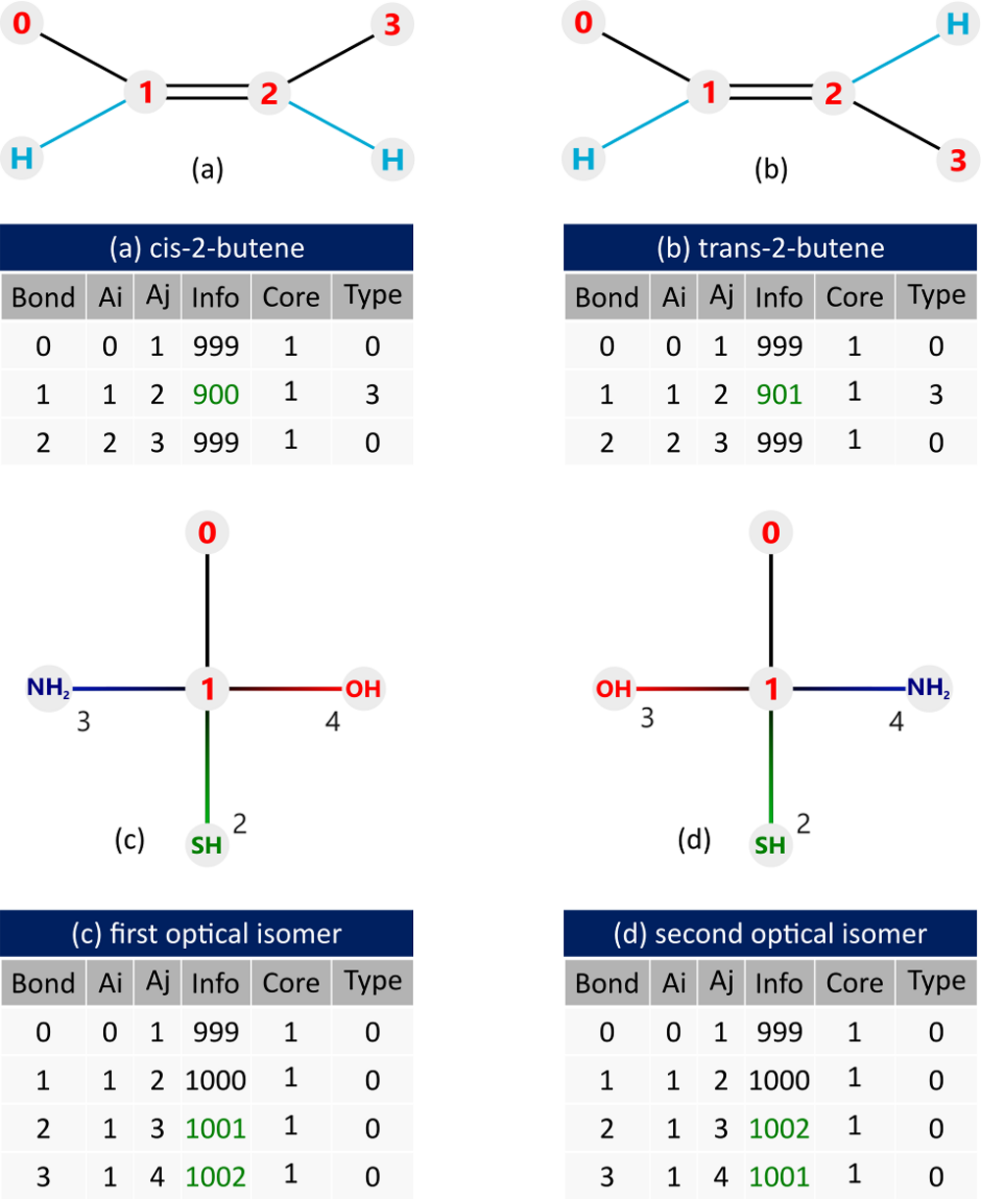
Examples of representation of isomers in the MAG matrix. Geometric isomers: (a) *cis*-2-butene. (b) *trans*-2-butene and (c) and (d) optical isomers.

To illustrate the differentiation of geometric isomers, Figure 12 presents the representations for (a) *cis*-2-butene and (b) *trans*-2-butene molecules. In these representations, the presence of codes 900 and 901 can be observed in the **Info** column for the row containing the double bond (**Type** = 3). The differentiation of optical isomers occurs by perceiving the order of bonds made by a quaternary carbon, provided that both molecules are numbered in the same order. In the example of Figure 12c,d, one can observe the different order in which the bonds appear in the molecule, enabling their differentiation.

## 4 Conclusions

The proposed methodology using the two matrices (MAG-SOLex) for simple and complex molecules can achieve a detailed molecular description and it can be easily communicated to different approaches for determining chemical reactions and properties for pure compounds. The application of this methodology to hydrocarbon mixtures, including those biomass-derived sources, demonstrates its effectiveness in providing a high level of molecular detail, enabling the identification of isomers and accurately predicting molecular properties using group contribution, and expected products using chemical reaction rules. Furthermore, the methodology allows for adapting other methods such as group contribution for property determination and molecules drawing, converting to the SMILES language. The adaptability of the methodology to different types of molecules and its wide range of potential applications make it a versatile and efficient approach for handling molecular information. New structural groups, bond types, and molecular structures can be easily added, demonstrating wide applicability in different areas of molecular analysis.

## Data Availability

The article data, along with the necessary files and usage instructions, are available at: https://github.com/pqgeunicamp/MAGSOLexArticlehttps://github.com/pqgeunicamp/MAGSOLexArticle. Additional data and details can be found in the Supporting Information.

## Supporting Information Available

Additional information related to this article, including detailed illustrations and comprehensive tables, can be found in the Supporting Information. Specifically, it includes: The Supporting Information is available free of charge at https://pubs.acs.org/doi/10.1021/acsomega.4c08940.

Stoichiometric matrix with the number of atoms in each molecule, complete MAG Matrices for complex molecules and Joback and Reid^23^ Table. Also, the SMILES coding for the molecules corresponding to, in order: Figure 2 – monocore molecule; Figure 3 – multicore molecule; Figure 4 – molecule example; Figure 5 – comparison between representations; Figure 7 – 4, 6-dimethyl-1-ethylnaphthalene; Figure 9 – reagent 0 and products 0 and 1 from reaction example; Figure 11 – isomeric oxygenated molecules (A–D); Figure 11 – geometric isomers (a–d)
Predicted and literature-obtained properties data for the comparative graphs (PDF)
