# Supplementary material for: MAG-SOLex Molecular Representation: A Methodology for Handling Complex Molecules in Algorithms
Source: ACS Omega. 2025 Feb 4;10(6):5645–58. doi: 10.1021/acsomega.4c08940 (PMC11840628; doi:10.1021/acsomega.4c08940)
Supplement: Supplementary file 1 — ao4c08940_si_001.pdf [file ao4c08940_si_001.pdf]

Supporting Information
MAG-SOLex molecular representation: a methodology for handling complex molecules in algorithms

Diego Telles Fernandes,[*,†] Karina Klock da Costa,[*,‡] Helton Siqueira Maciel,[*,†] Radha Liliane Pinto Gonçalves,[*,†] and Dirceu Noriler[*,‡]

†*PETROBRAS, Research and Development Center - CENPES. Av. Horacio de Macedo, 950, Ilha do Fundão, Rio de Janeiro, RJ, BR 20031-912*

‡*UNICAMP, School of Chemical Engineering. Albert Einstein Av, 500, Campinas, BR 13083-872*

E-mail: diegotelles@petrobras.com.br; karinakc@unicamp.br; helton.maciel@petrobras.com.br; radhag@petrobras.com.br; dnoriler@unicamp.br

1 Stoichiometric matrix

A matrix representing the number of atoms in each molecule can be derived from the SOLex representation. This is achieved by multiplying the zero position of the core dimension of the mixture's SOLex by the stoichiometric matrix (Table S1), as adapted from Quann and Jaffe[1] and detailed in the supporting information. The resulting matrix contains the quantities of C, H, S, N, O, Ni, and V atoms present in each molecule.

Table S1: Stoichiometric Matrix adapted from Quann and Jaffe[1]

	0	1	2	3	4	5	6	7	8	9	10	11	12	13	14	15	16	17	18	19	20
	A6	A4	A3	A2	N6	N5	4a	4n	3a	3m	3n	2n	2a	2m	2f	1a	1m	1n	Rp	Rm	Rn
C	6	4	3	2	6	5	4	4	3	3	3	2	2	2	2	1	1	1	1	1	0
H	6	2	1	0	12	10	6	6	4	4	4	2	2	2	2	0	0	0	3	2	-1
S	0	0	0	0	0	0	0	0	0	0	0	0	0	0	0	0	0	0	0	0	0
N	0	0	0	0	0	0	0	0	0	0	0	0	0	0	0	0	0	0	0	0	0
O	0	0	0	0	0	0	0	0	0	0	0	0	0	0	0	0	0	0	0	0	0
Ni	0	0	0	0	0	0	0	0	0	0	0	0	0	0	0	0	0	0	0	0	0
V	0	0	0	0	0	0	0	0	0	0	0	0	0	0	0	0	0	0	0	0	0

	21	22	23	24	25	26	27	28	29	30	31	32	33	34	35	36	37	38	39	40	41
	Ra	br	b2	Mn	Ma	Ho	Hn	Aa	Am	An	NS	RS	ANa	ANn	NN	RN	NO	RO	KO	Ni	VO
C	0	0	0	0	0	0	0	0	0	0	-1	0	-1	-1	-1	0	-1	0	0	0	0
H	-1	0	0	0	0	-2	-2	-2	-2	-2	-2	0	-1	-3	-1	1	-2	0	-2	-2	0
S	0	0	0	0	0	0	0	0	0	0	1	1	0	0	0	0	0	0	0	0	0
N	0	0	0	0	0	0	0	0	0	0	0	0	1	1	1	1	0	0	0	0	0
O	0	0	0	0	0	0	0	0	0	0	0	0	0	0	0	0	1	1	1	0	1
Ni	0	0	0	0	0	0	0	0	0	0	0	0	0	0	0	0	0	0	0	1	0
V	0	0	0	0	0	0	0	0	0	0	0	0	0	0	0	0	0	0	0	0	1

2 MAG Matrices for complex molecules

2.1 Monocore molecule

The monocore molecule is represented in Figure S1 where the first ring (**Info** = 0) is detailed from bonds 0 to 5: the connection between **Ai** = 0 and **Aj** = 1 is represented in the first row, there are six aromatic bonds (**Type** = 2) and a nitrogen atom (**Info** = 1001). The other ring bonds (6 to 33) are aromatic (**Type** = 2) or naphthenic (**Type** = 1). Bonds 34 to 37 represent methyl groups (indicated by **Info** = 999) connected to atoms previously enumerated on the rings. The main aliphatic chain is represented between bonds 38 and 54,

and its branches are shown between bonds 55 and 60. Note that the previously recommended atom and bond numbering is followed.

Bond	Ai	Aj	Info	Core	Type	Bond	Ai	Aj	Info	Core	Type	Bond	Ai	Aj	Info	Core	Type
0	0	1	0	1	2	21	16	18	4	1	1	41	34	35	999	1	0
1	1	2	1001	1	2	22	18	19	4	1	1	42	35	36	999	1	0
2	2	3	0	1	2	23	19	20	4	1	1	43	36	37	999	1	0
3	3	4	0	1	2	24	20	21	4	1	1	44	37	38	999	1	0
4	4	5	0	1	2	25	21	17	4	1	1	45	38	39	999	1	0
5	5	0	0	1	2	26	21	22	5	1	1	46	39	40	999	1	0
6	3	6	1	1	2	27	22	23	5	1	1	47	40	41	999	1	0
7	6	7	1	1	2	28	23	13	5	1	1	48	41	42	999	1	0
8	7	8	1	1	2	29	6	24	6	1	1	49	42	43	999	1	0
9	8	9	1	1	2	30	24	25	6	1	1	50	43	44	999	1	0
10	9	4	1	1	2	31	25	26	6	1	1	51	44	45	999	1	0
11	5	10	2	1	1	32	26	27	6	1	1	52	45	46	999	1	0
12	10	11	2	1	1	33	27	7	6	1	1	53	46	47	999	1	0
13	11	12	2	1	1	34	1	28	999	1	0	54	47	48	999	1	0
14	12	13	2	1	1	35	8	29	999	1	0	55	44	49	999	1	0
15	13	0	2	1	1	36	9	30	999	1	0	56	32	50	999	1	0
16	11	14	3	1	1	37	10	31	999	1	0	57	35	51	999	1	0
17	14	15	3	1	1	38	31	32	999	1	0	58	37	52	999	1	0
18	15	16	3	1	1	39	32	33	999	1	0	59	38	53	999	1	0
19	16	17	3	1	1	40	33	34	999	1	0	60	31	54	999	1	0
20	17	12	3	1	1												

Figure S1: MAG Matrix of a monocore molecule: Example 1.

2.2 Multicore molecule

The multicore molecule MAG matrix is presented in Figure S2. The first core (**Core** = 1) can be easily identified by the first set of ring bonds (0 to 24) and the first branched aliphatic chain (25 to 28). The second core (**Core** = 2) is identified by bonds 29 to 74, and the third core (**Core** = 3) by bonds 75 to 105. The aliphatics chains connecting the cores are represented in the **Core** column as 998 (bonds 106 to 114).

Bond	Ai	Aj	Info	Core	Type	Bond	Ai	Aj	Info	Core	Type	Bond	Ai	Aj	Info	Core	Type
0	0	1	0	1	2	39	34	29	6	2	2	77	70	71	9	3	2
1	1	2	0	1	2	40	30	35	7	2	1	78	71	72	9	3	2
2	2	3	0	1	2	41	35	36	7	2	1	79	72	73	9	3	2
3	3	4	0	1	2	42	36	37	7	2	1	80	73	68	9	3	2
4	4	5	0	1	2	43	37	38	7	2	1	81	73	74	10	3	2
5	5	0	0	1	2	44	38	25	7	2	1	82	74	75	10	3	2
6	1	6	2	1	1	45	31	39	8	2	1	83	75	76	10	3	2
7	6	7	1	1	2	46	39	40	8	2	1	84	76	77	10	3	2
8	7	8	1	1	2	47	40	41	8	2	1	85	77	68	10	3	2
9	8	9	1	1	2	48	41	42	8	2	1	86	71	78	11	3	2
10	9	10	1	1	2	49	42	32	8	2	1	87	78	79	11	3	2
11	10	11	1	1	2	50	26	43	999	2	0	88	79	80	11	3	2
12	11	6	1	1	2	51	27	44	999	2	0	89	80	81	11	3	2
13	2	12	1002	1	1	52	44	45	999	2	0	90	81	72	11	3	2
14	12	11	2	1	1	53	45	46	999	2	0	91	75	82	12	3	2
15	8	13	3	1	2	54	46	47	999	2	0	92	82	83	12	3	2
16	13	14	3	1	2	55	47	48	999	2	0	93	83	84	12	3	2
17	14	15	3	1	2	56	48	49	999	2	0	94	84	85	12	3	2
18	15	16	3	1	2	57	49	50	999	2	0	95	85	76	12	3	2
19	16	9	3	1	2	58	50	51	999	2	0	96	69	86	999	3	0
20	4	17	4	1	2	59	51	52	999	2	0	97	86	87	999	3	0
21	17	18	4	1	2	60	52	53	999	2	0	98	87	88	999	3	0
22	18	19	4	1	2	61	53	54	999	2	0	99	88	89	999	3	0
23	19	20	4	1	2	62	54	55	999	2	0	100	89	90	999	3	0
24	20	5	4	1	2	63	55	56	999	2	0	101	90	91	999	3	0
25	0	21	999	1	0	64	56	57	999	2	0	102	87	92	999	3	0
26	10	22	999	1	0	65	57	58	999	2	0	103	90	93	999	3	0
27	22	23	999	1	0	66	58	59	999	2	0	104	86	94	999	3	0
28	23	24	1001	1	0	67	59	60	999	2	0	105	91	95	1001	3	0
29	25	26	5	2	2	68	60	61	999	2	0	106	13	96	999	998	0
30	26	27	5	2	2	69	61	62	999	2	0	107	96	97	999	998	0
31	27	28	5	2	2	70	62	63	999	2	0	108	97	98	999	998	0
32	28	29	5	2	2	71	63	64	999	2	0	109	98	99	999	998	0
33	29	30	5	2	2	72	64	65	999	2	0	110	99	33	999	998	0
34	30	25	5	2	2	73	65	66	999	2	0	111	14	100	999	998	0
35	28	31	6	2	2	74	66	67	1002	2	0	112	100	101	999	998	0
36	31	32	6	2	2	75	68	69	9	3	2	113	101	102	999	998	0
37	32	33	6	2	2	76	69	70	9	3	2	114	102	70	999	998	0
38	33	34	6	2	2												

Figure S2: MAG Matrix of a multicore molecule: Example 2.

3 Joback Table

One of the simplest group contribution methods for calculating thermodynamic properties is the Joback and Reid[2] method. It is possible to obtain a matrix of Joback groups using only the SOLex representation, which was one of Maciel[3]'s original objectives when expanding SOL. This is achieved through matrix multiplication between the Table S2 and the zero position of the core dimension of SOLex of the mixture, excluding the **RCn** and **Arr** columns. The Joback Table presents the relationship between the SOLex attributes and the 23 Joback groups that Maciel used to represent the molecules in his molecular reconstruction problem. This matrix multiplication results in a matrix with molecules in the rows and Joback groups in the columns, thereby enabling the calculation of properties of pure components such as critical temperature, critical pressure, acentric factor, ideal-gas heat capacity, liquid viscosity, boiling point, melting point, enthalpy of vaporization, enthalpy of formation, and Gibbs free energy of formation.

**Joback groups**	0 **A6**	1 **A4**	2 **A3**	3 **A2**	4 **N6**	5 **N5**	6 **N4a**	7 **N4n**	8 **N3a**	9 **N3m**	10 **N3n**	11 **N2n**	12 **N2a**	13 **N2m**	14 **N2f**	15 **N1a**	16 **N1m**	17 **N1n**	18 **Rp**	19 **Rm**	20 **Rn**	21 **Ra**	22 **br**	23 **br2**	24 **MEn**	25 **MEa**	26 **IHo**	27 **IHn**	28 **AAa**	29 **AAm**	30 **AAn**	31 **NS**	32 **RS**	33 **ANa**	34 **ANn**	35 **NN**	36 **RN**	37 **NO**	38 **RO**	39 **KO**	40 **Ni**	41 **VO**
$-CH_3$ *nr* aliphatic CH_3	0	0	0	0	0	0	0	0	0	0	0	0	0	0	0	0	0	0	1	0	0	0	1	1	1	1	0	0	0	0	0	0	0	0	0	0	0	0	0	0	0	0
$-CH_2-$ *nr* aliphatic CH_2	0	0	0	0	0	0	0	0	0	0	0	0	0	0	0	0	0	0	0	1	0	0	-2	-1	-1	-1	2	0	0	0	0	0	0	0	0	0	0	0	0	-1	0	0
$-CH_2-$ *r* naphthenic CH_2	0	0	0	0	6	5	4	2	3	2	1	0	2	1	1	1	0	-1	0	0	-1	0	0	0	-1	0	0	2	0	-1	-2	-1	0	0	-2	-1	0	-1	0	0	0	0
>CH- *nr* aliphatic CH	0	0	0	0	0	0	0	0	0	0	0	0	0	0	0	0	0	0	0	0	0	0	1	-1	0	0	0	0	0	0	0	0	0	0	0	0	0	0	0	0	0	0
>CH- r_1 condensed naphthenic	0	0	0	0	0	0	0	2	0	1	2	2	0	1	1	0	1	2	0	0	0	0	0	0	0	0	0	0	0	1	2	0	0	0	0	0	0	0	0	0	0	0
>CH- r_2 substituted naphthenic	0	0	0	0	0	0	0	0	0	0	0	0	0	0	0	0	0	0	0	0	1	0	0	0	1	0	0	0	0	0	0	0	0	0	0	0	0	0	0	0	0	0
=CH- *nr* linear olefin	0	0	0	0	0	0	0	0	0	0	0	0	0	0	0	0	0	0	0	0	0	0	0	0	0	0	-2	0	0	0	0	0	0	0	0	0	0	0	0	0	0	0
=CH- *r* cyclic olefin	0	0	0	0	0	0	0	0	0	0	0	0	0	0	0	0	0	0	0	0	0	0	0	0	0	0	0	-2	0	0	0	0	0	0	1	0	0	0	0	0	0	0
aCH *r* aromatic CH	6	2	1	0	0	0	-2	0	-2	-1	0	0	-2	-1	-1	-2	-1	0	0	0	0	-1	0	0	0	-1	0	0	-2	-1	0	0	0	-1	0	0	0	0	0	0	0	0
aC r_1 peripheral condensed aromatic	0	2	2	0	0	0	2	0	2	1	0	0	0	0	-1	2	1	0	0	0	0	0	0	0	0	0	0	0	2	1	0	0	0	0	0	0	0	0	0	0	0	0
aC r_2 internal condensed aromatic	0	0	0	2	0	0	0	0	0	0	0	0	2	1	2	0	0	0	0	0	0	0	0	0	0	0	0	0	0	0	0	0	0	0	0	0	0	0	0	0	0	0
aC- r_3 substituted aromatic	0	0	0	0	0	0	0	0	0	0	0	0	0	0	0	0	0	0	0	0	0	1	0	0	0	1	0	0	0	0	0	0	0	0	0	0	0	0	0	0	0	0
-S- *r* aromatic sulfur	0	0	0	0	0	0	0	0	0	0	0	0	0	0	0	0	0	0	0	0	0	0	0	0	0	0	0	0	0	0	0	1	0	0	0	0	0	0	0	0	0	0
-S- *nr* aliphatic sulfur	0	0	0	0	0	0	0	0	0	0	0	0	0	0	0	0	0	0	0	0	0	0	0	0	0	0	0	0	0	0	0	0	1	0	0	0	0	0	0	0	0	0
=N- *r* piridinic nitrogen	0	0	0	0	0	0	0	0	0	0	0	0	0	0	0	0	0	0	0	0	0	0	0	0	0	0	0	0	0	0	0	0	0	1	1	0	0	0	0	0	0	0
-NH- *r* pyrrolic nitrogen	0	0	0	0	0	0	0	0	0	0	0	0	0	0	0	0	0	0	0	0	0	0	0	0	0	0	0	0	0	0	0	0	0	0	0	1	0	0	0	0	0	0
-NH- *nr* aliphatic nitrogen	0	0	0	0	0	0	0	0	0	0	0	0	0	0	0	0	0	0	0	0	0	0	0	0	0	0	0	0	0	0	0	0	0	0	0	0	1	0	0	0	0	0
-O- *r* aromatic oxygen	0	0	0	0	0	0	0	0	0	0	0	0	0	0	0	0	0	0	0	0	0	0	0	0	0	0	0	0	0	0	0	0	0	0	0	0	0	1	0	0	0	0
-O- *nr* aliphatic oxygen	0	0	0	0	0	0	0	0	0	0	0	0	0	0	0	0	0	0	0	0	0	0	0	0	0	0	0	0	0	0	0	0	0	0	0	0	0	0	1	0	0	0
C=O *nr* carbonyl	0	0	0	0	0	0	0	0	0	0	0	0	0	0	0	0	0	0	0	0	0	0	0	0	0	0	0	0	0	0	0	0	0	0	0	0	0	0	0	1	0	0
-Ni- nickel	0	0	0	0	0	0	0	0	0	0	0	0	0	0	0	0	0	0	0	0	0	0	0	0	0	0	0	0	0	0	0	0	0	0	0	0	0	0	0	0	1	0
-VO- vanadium oxide	0	0	0	0	0	0	0	0	0	0	0	0	0	0	0	0	0	0	0	0	0	0	0	0	0	0	0	0	0	0	0	0	0	0	0	0	0	0	0	0	0	1
>C< aliphatic C	0	0	0	0	0	0	0	0	0	0	0	0	0	0	0	0	0	0	0	0	0	0	0	1	0	0	0	0	0	0	0	0	0	0	0	0	0	0	0	0	0	0
**Overall Balance**	**6**	**4**	**3**	**2**	**6**	**5**	**4**	**4**	**3**	**3**	**3**	**2**	**2**	**2**	**2**	**1**	**1**	**1**	**1**	**1**	**0**	**0**	**0**	**0**	**0**	**0**	**0**	**0**	**0**	**0**	**0**	**0**	**1**	**0**	**0**	**0**	**1**	**0**	**1**	**0**	**1**	**1**

Table S2: Joback and Reid[2] Groups matrix.

4 SMILES Table

The SMILES representation of the molecules discussed in this article is provided in Table S3 in the order of their appearance in the text for clarity and reproducibility. This notation enables the concise depiction of chemical structures and facilitates their use in computational analysis and chemical tools.

Table S3: SMILES representation of the molecules

Number	Smile representation
1	Cc1=c2-c(C(C(C)C(C)CCC(C)CC(C)C(C)CCCCCC(C)CCCC)C3C4C2CCC2 C4C(CCC2)CC3)=[c]=2-[c](=c3-c(CCCC3)=c(C)-c2C)=N1
2	NCCc=1-[c]=2=[c](=c(CCCCc-3=c4-c(CCCC4)=[c]-4=c(CCCCCCCCCCC CCCCCCCCCCCCO)-c(C)=c5-c(CCCC5)=[c]4=c3)-c(CCCc=3-c(C(C)C(C)CC C(C)CN)=[c]=4-[c](=c-[c]=5=[c](=c-c=c-c5)-c4)=c-4-c3-c=c-c=c4)=c-c2)-c=c -2-c-3=c(C)-c-4=c(-c=c-c=c4)-c=c3Oc12
3	CCC(C)C1Cc-2=c-c=c-c=c2CC1
4	NCC(C)CC1C2c-3=c(CCC2)-c=c-c=c3CC1
5	CCc1ccc(C)c2cc(C)ccc12
6	CCC(C)C2CCc1ccccc1C2
7	CC2CCC1CCCCC1C2
8	CCC
9	O=CC(O)CC1CCCCC1
10	O=C(CO)CC1CCCCC1
11	O=CCOCC1CCCCC1
12	O=C(O)CCC1CCCCC1
13	C/C=C\C
14	C/C=C\C
15	CC(N)(O)S
16	CC(N)(O)S

5 Data comparison

The data used to obtain Figure 8 from the original article are detailed in TablesS4 and S4.

nº	Molecule		Literature			Predicted			
			**Pc [bar]**	**Tc [K]**	**Tb [K]**	**Pc[bar]**	**Tc[K]**	**TbMG[K]**	**TbPR[K] EOS**
0	anthracene	C14H10	29,0	873,0	615,2	32,05	866,1	607	588,1
1	1,3,8-trimethylnaphthalene	C13H14	26,7	786,8	558,2	25,83	790,9	555,9	543,5
2	dibenzofuran	C12H8O	32,0	837,8	557,9	28,49	812,2	573,9	552,8
3	dibenzopyrrole	C12H9N	32,6	899,0	627,9	30,62	852	601,5	596,1
4	1-tridecanal	C13H26O	17,4	700,0	540,2	18,14	717	538,5	508,5
5	4,6-dimethyl-1-ethylnaphthalene	C14H16	24,2	811,4	585,4	24,73	806,7	572,8	558,1
6	trans-1,3-dimethylcyclohexane	C8H16	29,4	598,0	397,6	30,75	597,2	405,4	389,3
7	butylcyclohexane	C10H20	25,7	667,0	454,1	25,69	644,8	454,3	434,3
8	2-butylnaphthalene	C14H16	25,0	781,0	562,2	24,77	805,3	572,3	555,5
9	pentatriacontylbenzene	C41H76	4,3	1048,4	801,2	8,51	942,1	757,6	752,5
10	pentatriacontylcyclohexane	C41H82	4,1	1060,8	807,2	8,38	937,2	755,7	750,3
11	pentatriacontane	C35H72	4,8	968,3	763,2	8,82	892,7	718,7	703
12	hexadecane	C16H34	14,2	720,6	560,0	14,85	716,4	547,3	515,6
13	N-heptacontane	C70H142	1,6	7326,5	926,2	6,86	1050,9	871,7	879,2
14	benzene	C6H6	49,2	562,1	353,2	48,95	578,5	359	358,9
15	toluene	C7H8	41,0	591,8	383,8	42,18	604,1	389,2	382,5
16	n-heptane	C7H16	27,4	540,2	371,6	27,83	536,2	369,8	351,7
17	cyclohexene	C6H12	40,5	553,2	353,9	41,29	560,5	355,5	358,3
18	methyl-cyclohexane	C7H14	34,8	572,1	374,1	35,32	579,6	381,8	374,3
19	2,3-dimethylpentane	C7H16	29,1	537,3	362,9	28,33	521,2	344,2	345,3
20	n-hexano	C6H14	29,9	507,9	341,9	30,88	503,7	337,4	325,7
20	propanol	C3H8O	47,6	508,3	355,4	56,41	500,8	322,8	321

Table S4: Literature vs Predicted Data: Pc [bar], Tc [K], Tb [K]

Table S5: Literature vs Predicted Data: Hf [kJ/mol]

**Molecule**	**Hf kJ/mol @ 298 K**	
	**Literature**	**Predicted**
anthracene	227,7	214,907
1,3,8-trimethylnaphthalene	57	56,968
dibenzofuran	83,4	80,757
dibenzopyrrole	209,6	298,146
1-tridecanal	-394,1	-396,865
4,6-dimethyl-1-ethylnaphthalene	51,3	33,613
trans-1,3-dimethylcyclohexane	-176,5	-178,637
butylcyclohexane	-213,17	-204,756
2-butylnaphthalene	52,3	56,193
pentatriacontylbenzene	-653,04	-655,602
pentatriacontylcyclohexane	-835,25	-850,455
pentatriacontane	-765,73	-766,766
hexadecane	-374,17	-371,015
N-heptacontane	-1488,1	-1495,78

References

(1) Quann, R. J.; Jaffe, S. B. Structure-Oriented Lumping: Describing the Chemistry of Complex Hydrocarbon Mixtures. *Ind. Eng. Chem. Res.* **1992**, *31*, 2483–2497.

(2) Joback, K. G.; Reid, R. C. Estimation of Pure-Component Properties from Group-Contributions. *Chemical Engineering Communications* **1987**, *57*, 233–243.

(3) Maciel, H. S. Molecular Reconstruction of Heavy Petroleum Fractions. M.Sc. thesis, UFRJ, Rio de Janeiro, Brazil, 2019.
